# Unraveling the relationships among pandemic fear, cyberchondria, and alexithymia after China’s exit from the zero-COVID policy: insights from a multi-center network analysis

**DOI:** 10.3389/fpsyt.2024.1489961

**Published:** 2024-11-14

**Authors:** Yuan Li, Jie Li, Chunfen Zhou, Chuanya Huang, Biru Luo, Yanling Hu, Xi Huang, Jinbo Fang

**Affiliations:** ^1^ Department of Nursing, West China Second University Hospital, Sichuan University, Chengdu, China; ^2^ Department of Neonatology, West China Second University Hospital, Sichuan University, Chengdu, China; ^3^ Key Laboratory of Birth Defects and Related Diseases of Women and Children (Sichuan University), Ministry of Education, Chengdu, China; ^4^ Mental Health Center, West China Hospital, Sichuan University, Chengdu, China; ^5^ West China School of Nursing/West China Hospital, Sichuan University, Chengdu, China

**Keywords:** pandemic fear, cyberchondria, alexithymia, COVID-19, network analysis, nurse

## Abstract

**Objective:**

China’s abrupt exit from the zero-COVID policy in late 2022 led to a rapid surge in infections, overwhelming healthcare systems and exposing healthcare providers to intensified psychological pressures. This sudden shift exacerbated pandemic-related psychological issues, including fear, health anxiety, and emotional processing difficulties. This study aimed to unravel the relationships among pandemic fear, cyberchondria, and alexithymia following China’s exit from the zero-COVID policy.

**Methods:**

A multi-center cross-sectional survey was conducted among 4088 nurses from 43 public hospitals in China. The web-based survey comprised the Fear of COVID-19 Scale, Cyberchondria Severity Scale, and Toronto Alexithymia Scale. Network analysis was employed to explore the interconnections and identify central components within these psychological and behavioral constructs.

**Results:**

The analysis revealed a dense network with predominantly positive connections. Specific aspects of cyberchondria and pandemic fear exhibited the highest strength centrality, indicating their critical influence. The externally oriented thinking dimension of alexithymia emerged as a crucial bridge node, linking pandemic fear and cyberchondria. The network structure demonstrated consistency across diverse educational backgrounds and career stages.

**Conclusion:**

These findings highlight the need for targeted interventions focusing on key network components, particularly externally oriented thinking, to disrupt the detrimental cycle of pandemic fear and cyberchondria. Healthcare organizations should promote balanced objective fact-focused and problem-solving approaches while also fostering skills in emotional awareness and expression, thereby mitigating the risk of maladaptive pandemic fear responses and dysfunctional online health information-seeking behaviors.

## 1 Introduction

The COVID-19 pandemic has dramatically reshaped the global health landscape, with its repercussions reverberating through physical and psychological domains of human life (1–3). China’s pandemic management strategy diverged markedly from global norms, implementing stringent containment measures collectively known as the “zero-COVID” policy. This approach involved mass testing, strict quarantines, and rapid lockdowns upon case detection, effectively suppressing viral transmission for nearly three years (4). Nevertheless, December 2022 marked a pivotal moment as China rapidly pivoted away from this strategy, signaling a substantial shift in its pandemic response (4). The abrupt end to the zero-COVID policy, while representing a critical transition, exposed a population with limited herd immunity to an unprecedented viral surge (5), resulting in a rapid escalation of infection rates and widespread public concern. During this transition period, an estimated 97% of the population (1.37 billion people) became infected, pushing the healthcare system to its limits (6).

In addition to a spike in COVID-19 cases, the sudden policy pivot also exacerbated pandemic-related fear. A scoping review reported that up to 45.2% of the general population experienced moderate to severe levels of fear prior to the policy change (7). Following the strategy shift, this prevalence became more pronounced, especially among healthcare workers, with a nation-wide investigation conducted immediately after the policy change revealing that 60.8% of healthcare professionals reported significant COVID-19 fear (8). Pandemic fear is conceptualized as apprehension about either being infected or infecting others, and involves alarm reactions that trigger a cascade of physiological, cognitive, and behavioral changes (7, 9, 10). When modulated appropriately, this fear can foster adaptive practices such as adherence to preventive measures (11). However, sustained high levels of fear potentially impair cognitive processing and rational decision-making in response to COVID-19 (9), manifesting as adverse psychological reactions (e.g., anxiety, avoidance, and aversion), and even socially disruptive behaviors (e.g., panic buying, interpersonal conflicts, and dissemination of COVID-19 misinformation) (11–13). Among healthcare professionals, these negative impacts can lead to decreased work satisfaction, increased absenteeism, and ultimately, deterioration in the quality of healthcare service provision (8, 14, 15). In this regard, COVID-19 fear not only affects individual well-being but also poses significant challenges to the resilience and effectiveness of healthcare systems.

The pervasive pandemic fear, coupled with the ubiquity of digital information, has given rise to another concerning phenomenon: cyberchondria, defined as a pathological compulsion driven by overly health anxiety that results in obsessive online health information searching behaviors (16). During the uncertain period, a surge in cyberchondria prevalence was observed (17, 18), with COVID-19-related fear being identified as the strongest predictor of cyberchondria-related distress (19, 20). Data from the China Internet Network Information Center showed a steady growth in online medical users since the end of the dynamic zero-COVID policy (21). A systematic review reported that approximately 79.0% of the population in mainland China engages in online health information seeking (22), with nearly half of these individuals experiencing increased health anxiety as a consequence (23). This excessive or repeated health-related information seeking may, in turn, amplify pandemic fear and exacerbate psychopathological vulnerabilities (20, 24), leading to a detrimental cycle where individuals persistently seek more information to alleviate their concerns, paradoxically resulting in elevated levels of fear and anxiety. In the context of healthcare professionals, who face heightened infection risks and greater fear and anxiety, the susceptibility for cyberchondria is particularly concerning (18, 25).

Alexithymia refers to a personality trait characterized by deficits in identifying and expressing emotions, as well as a tendency toward externally oriented thinking (26). It occurs in approximately 10% of the general population (27) but is substantially more prevalent among healthcare professionals, particularly nurses (28–30). Unresolved emotional conflicts and stress can accumulate over time, manifesting in a spectrum of maladaptive response patterns, including withdrawal, self-blame, excessive apprehension, and obsessive-compulsive disorders (31, 32). A recent study has unveiled a strong correlation between alexithymia and cyberchondria, indicating that alexithymic individuals may be more prone to excessive online health information seeking (33). Moreover, another study has identified alexithymia as a potential transdiagnostic mechanism linking pandemic fear and cyberchondria (18). These findings are especially pertinent given the heightened stress and emotional demands placed on healthcare professionals amid the policy shift.

Despite emerging research on pandemic fear, cyberchondria, and alexithymia, most studies have examined these constructs in isolation or through simple correlational analyses (18, 19, 33). There remains a need to further explore the complex relationships among these psychological traits and health-related behaviors, particularly in the unique context of China’s dramatic policy shift, to inform the development of more precise and individualized interventions for healthcare professionals experiencing pandemic-related psychological distress. Network analysis offers a promising avenue to elucidate these complex relationships (34). This method models psychological and behavioral constructs as systems of interconnected components, with nodes representing variables (e.g., symptoms, behaviors, traits) and edges depicting the relationships between them. Network analysis allows for the identification of central nodes—highly connected variables that may play pivotal roles within the network—and bridge nodes that link different clusters of variables, indicating pathways through which constructs influence one another (34). By conceptualizing psychological and behavioral constructs in this manner, network analysis can reveal the strength and patterns of connections between specific items and dimensions of pandemic fear, cyberchondria, and alexithymia, providing a more in-depth understanding of their mutual influences and informing key intervention targets and pathways for mitigating psychological distress and promoting adaptive health behaviors.

The present study aimed to investigate the complex relationships among these constructs in nurses following China’s exit from the zero-COVID policy. Specifically, we sought to explore how the individual items of pandemic fear and cyberchondria, along with the dimensions of alexithymia, interact and potentially reinforce each other. Additionally, we aimed to identify the central and bridge components within the network of these psychological and behavioral constructs.

## 2 Methods

### 2.1 Study design and participants

This study utilized a multicenter cross-sectional design, adhering to the Strengthening the Reporting of Observational Studies in Epidemiology (STROBE) Statement for cross-sectional studies (35). Participants were recruited using a convenience sampling method from forty-three public hospitals, primarily located in China’s western and central regions. These areas, compared to the eastern and southern parts of the country, are not only less developed economically but also face significant challenges in healthcare resource allocation, with fewer advanced medical facilities and healthcare professionals per capita (36). Public hospitals in China, serving as the backbone of the nation’s healthcare system, were pivotal during every wave of the pandemic outbreak and remain crucial in managing health concerns in the post-pandemic era (37). Nurses in these institutions have been at the forefront of addressing pandemic-related challenges, making them ideal target population for examining the relationships among pandemic fear, cyberchondria, and alexithymia (10, 38). Eligible participants included active registered nurses with more than one year of clinical experience who voluntarily consented to participate. The study excluded nurses on rotation assignments, those in internship roles, and individuals who had taken continuous leave exceeding six months within the past year.

### 2.2 Measures

#### 2.2.1 Sociodemographic information

Sociodemographic data from the nurse participants were collected including information on gender, age, marital status, highest educational attainment, years of working experience, and professional title. Participants were also asked if they had previously contracted COVID-19.

#### 2.2.2 Pandemic fear

Pandemic fear was assessed using the Fear of COVID-19 Scale (FCV-19S) (9). This 7-item scale comprises three items related to physical responses (i.e., FCV.3, FCV.6, and FCV.7) and four items addressing psychological responses to COVID-19 (i.e., FCV.1, FCV.2, FCV.4, and FCV.5) (11). Participants rated each item on a 5-point Likert scale (1 = “strongly disagree” to 5 = “strongly agree”). The total score is calculated by summing the individual item scores, resulting in a range from 7 to 35, with higher scores indicating greater fear of COVID-19. A sample item is: “My heart races or palpitates when I think about getting coronavirus-19”. The scale has demonstrated good psychometric properties in Chinese populations (39). In the present study, the FCV-19S exhibited excellent internal consistency, evidenced by a Cronbach’s alpha coefficient of 0.963 and a Guttman split-half reliability of 0.908.

#### 2.2.3 Cyberchondria

Cyberchondria was measured using the Short-Form Version of the Cyberchondria Severity Scale (CSS-12) (40). This 12-item scale employs a 5-point Likert scale (1 = “never” to 5 = “always”). The cumulative score, ranging from 12 to 60, is calculated by summing the scores for all 12 items, with higher scores indicating more severe cyberchondria. A representative item reads: “If I notice an unexplained bodily sensation, I will search for it on the internet”. The CSS-12 has demonstrated good reliability and validity globally, including within the Chinese population (18, 41). In the current study, the scale’s reliability was high, with a Cronbach’s alpha of 0.974 and a Guttman’s split-half reliability of 0.927.

#### 2.2.4 Alexithymia

Alexithymia was measured using the Toronto 20-item Alexithymia Scale (TAS-20) (42, 43). This scale evaluates three established dimensions: difficulty identifying feelings (TAS.D1), difficulty describing feelings (TAS.D2), and externally oriented thinking (TAS.D3). Each item is rated on a 5-point Likert scale (1 = “totally disagree” to 5 = “totally agree”), with the total score ranging from 20 to 100, representing the sum of all 20 items. Higher scores indicate more significant alexithymia-related issues. An example item is: “I prefer talking to people about their daily activities rather than their feelings.” The Chinese version of the TAS-20 has been verified to have adequate reliability and validity (44). In this study, the scale showed good overall reliability (Cronbach’s alpha = 0.831). The Cronbach’s alpha coefficients for the subscales were as follows: 0.962 for difficulty identifying feelings, 0.897 for difficulty describing feelings, and 0.777 for externally oriented thinking.

### 2.3 Data collection

The study collected data from January to April 2023, immediately following China’s cessation of the zero-COVID strategy. The principal investigator initially secured consent from nursing department heads at selected hospitals, who subsequently disseminated an e-flyer containing a WeChat QR code to potential participants within their institutions. This code directed nurses to the questionnaire hosted on the Survey Star platform (www.wjx.cn). Upon accessing the questionnaire, participants were presented with an introductory interface detailing the study’s objectives, completion instructions, and measures ensuring respondent privacy and confidentiality. Nurses who wished to participate were required to provide electronic informed consent before proceeding to the questionnaire. The platform was configured to accept only one submission per IP address and employed a paginated format requiring full completion of each section.

### 2.4 Statistical analysis

All statistical analyses were performed using R version 4.3.2. Descriptive statistics were computed for all variables; continuous variables were presented as means ± standard deviations (SD), while categorical variables were expressed as frequencies and percentages. Pearson correlation analyses were conducted to provide an initial overview of relationships between the 7 items of pandemic fear (FCV-19S), 3 dimensions of alexithymia (TAS-20), and 12 items of cyberchondria (CSS-12).

#### 2.4.1 Network estimation

To explore the complex relationships among pandemic fear, cyberchondria, and alexithymia, we constructed undirected networks using the *qgraph* package in R. The network estimation employed regularized partial correlation analyses (45) with the least absolute shrinkage and selection operator (LASSO), combined with the extended Bayesian information criterion (EBIC) for model optimization (46, 47). The network visualization utilized the Fruchterman-Reingold algorithm with a “spring” layout, positioning more influential nodes centrally and strongly connected nodes in closer proximity (48).

In the resultant undirected network, nodes represent individual variables derived from the measured psychological constructs: 7 items of pandemic fear (FCV-19S), 3 dimensions of alexithymia (TAS-20), and 12 items of cyberchondria (CSS-12). To maintain a parsimonious network structure while preserving construct validity, we utilized the three established dimensions of the TAS-20 instead of individual items (42, 43). The rationale for combining item-level and dimension-level data within a single network analysis is as follows. First, the dimensions of alexithymia represent distinct theoretical constructs, serving as meaningful nodes in the network, while the items within FCV-19S (9) and CSS-12 (40) exhibit closer intra-scale relationships, warranting item-level analysis. Second, this approach mitigates the risk of an overly complex network structure that could potentially obscure key relationships. Finally, it respects the psychometric properties and established factor structure of the TAS-20 while allowing for a more granular examination of the relatively newer FCV-19S (9) and CSS-12 scales (40). In the network visualization, edges denote partial correlations between nodes, with edge thickness proportional to the strength of association; blue edges signify positive correlations, whereas red edges represent negative correlations (48).

#### 2.4.2 Centrality and bridge centrality analyses

Centrality analyses were conducted using the *qgraph* package to identify core constructs within the network from a mechanistic perspective, focusing on how different elements interact and influence each other. We computed three common centrality indices: strength, closeness, and betweenness (34, 49). Strength centrality, defined as the sum of absolute edge weights connected to a node, represents a construct’s potential influence on other network elements (46). Closeness centrality, the inverse of the sum of shortest paths to all other nodes, indicates the core position of the construct in the network, while betweenness centrality measures the frequency of a node lying on the shortest path between other nodes, highlighting the construct’s bridging function (46, 49). However, given that closeness and betweenness centralities are often less reliably estimated in psychological networks, we primarily focused on strength centrality for interpretation, as it is considered the most reliable and interpretable index (46, 49).

Additionally, we utilized the *networktools* package to identify bridge constructs, which may serve as critical links among pandemic fear, cyberchondria, and alexithymia clusters. In this study, bridge constructs, defined as nodes connecting different construct clusters, were identified using bridge strength centrality (50, 51). This metric quantifies a node’s potential to influence or be influenced by nodes in other clusters (52).

#### 2.4.3 Network accuracy and stability

We employed bootstrapping methods using the *bootnet* package in R to assess the network’s accuracy and stability. First, we conducted non-parametric bootstrapping (nBoots = 500) to estimate the accuracy of edge-weights by computing 95% confidence intervals (CIs) (45). Second, to evaluate the network stability, we performed a case-dropping subset bootstrap procedure (nBoots = 500) to compute the correlation stability (CS) coefficient. The CS-coefficient indicates the maximum proportion of cases that can be dropped while maintaining a correlation of at least 0.7 between original centrality indices and those of the subset networks, with 95% probability (53). A CS-coefficient should exceed 0.25 and preferably surpass 0.5 for robust stability (46). Finally, we undertook bootstrapped difference tests to evaluate the statistical significance of differences in network properties, specifically focusing on edge weights, node strengths, and node bridge strengths (46).

#### 2.4.4 Network comparisons

To investigate differences in network structure and properties between subgroups, we employed the *NetworkComparisonTest* (NCT) package. NCT is a two-tailed permutation test that evaluates differences in global strength, network structure, and edge strength between networks (54). Statistical significance was set at *P* < 0.05. Recognizing that NCT’s statistical power can be compromised by unequal sample sizes (55), we strategically selected subgroups to ensure balanced comparisons. Based on the characteristics and data distribution of our study population, we conducted two primary subgroup analyses stratified by educational level and working experience. For each comparison, we performed 1000 permutations to assess global network strength (the absolute sum of all edge weights) and network structure (the distribution of edge weights) (54). Furthermore, we evaluated individual edge strengths between networks using Holm-Bonferroni corrected comparisons to account for multiple testing (46). This approach allowed us to identify both overall structural differences and specific edge disparities between subgroups (54).

## 3 Results

Our online survey garnered 4088 submissions. After excluding responses completed in less than 3 minutes (as the questionnaire required a minimum of 3 minutes to complete) or those showing consistent response patterns, 3977 valid responses were retained, yielding an effective response rate of 97.3%. In network analysis, sample sizes exceeding 1,000 are considered sufficient to generate reliable network estimates (46); therefore, our sample size satisfies this requirement and enhances the reliability of our findings.

### 3.1 Participant characteristics and the measured constructs

**Table 1** summarizes the participants’ characteristics. The nurses’ mean age was 33.3 ± 7.0 years, with a predominantly female (95.2%) and married (71.8%) composition. The highest educational level attained was junior college or below for 44.3% and undergraduate degree or above for 55.7%. Participants averaged 11.3 ± 7.6 years of working experience, with a relatively balanced distribution across experience levels. The majority held junior professional titles (72.0%), and a significant proportion (85.0%) reported prior COVID-19 infection. Additionally, most participants (86.4%) self-reported their socioeconomic status as lower or middle tier.

**Table 1:** Demographics of the participating nurses (N = 3977).

Characteristics	n (%)/Mean ± SD^*^
Gender	
Male	191 (4.8%)
Female	3786 (95.2%)
Age (years)	33.3 ± 7.0
≤ 28	1037 (26.1%)
29-36	1951 (49.1%)
37-44	642 (16.1%)
≥ 45	347 (8.7%)
Marital status^†^	
Single	1121 (28.2%)
Married	2856 (71.8%)
Educational level	
Junior college or below	1761 (44.3%)
Undergraduate or above	2216 (55.7%)
Working experience (years)	11.3 ± 7.6
1-5	980 (24.6%)
6-10	1033 (26.0%)
11-15	1128 (28.4%)
≥ 16	836 (21.0%)
Professional title	
Junior	2865 (72.0%)
Intermediate	908 (22.8%)
Senior	204 (5.1%)
COVID-19 infection	
Infected	3382 (85.0%)
Not infected	595 (15.0%)

^*^SD, standard deviation.

^†^Single indicated separated, divorced, widowed, or never married, and married indicated married or partnered.

**Table 2** presents detailed descriptive statistics for the three main constructs of this study: pandemic fear (7 items), cyberchondria (12 items), and alexithymia (3 dimensions). In addition, **Supplementary Table S1** and **Supplementary Figure S1** provided the results of Pearson correlation analyses for the three constructs. Significant correlations were observed between almost all variables, providing context for the subsequent network analysis and highlighting the complex interrelationships among the studied constructs.

**Table 2:** Descriptive statistics for pandemic fear, cyberchondria, and alexithymia (N = 3977).

Variables (measures: range)	Mean ± SD^*^
Pandemic fear (FCV-19S: 7 to 35)	15.61 ± 7.00
FCV.1: Afraid of COVID-19	2.36 ± 1.13
FCV.2: Discomfort when thinking about COVID-19	2.37 ± 1.16
FCV.3: Clammy hands when thinking about COVID-19	2.13 ± 1.06
FCV.4: Fear of losing life due to COVID-19	2.23 ± 1.13
FCV.5: Nervousness when watching news about COVID-19	2.28 ± 1.12
FCV.6: Sleep difficulties due to worry about COVID-19	2.12 ± 1.07
FCV.7: Palpitations when thinking about COVID-19	2.11 ± 1.06
Cyberchondria (CSS-12: 12 to 60)	25.30 ± 11.28
CSS.1: Search unexplained sensation online	2.44 ± 1.09
CSS.2: Symptom research distracts from online activities	2.24 ± 1.07
CSS.3: Read various web pages on symptoms	2.19 ± 1.07
CSS.4: Panic reading rare online conditions	2.15 ± 1.10
CSS.5: Online research leads to consultation	2.25 ± 1.10
CSS.6: Repeatedly search symptoms online	2.15 ± 1.07
CSS.7: Symptom research interrupts work	1.95 ± 1.04
CSS.8: Fine until reading serious conditions online	1.98 ± 1.03
CSS.9: Increased anxiety after symptom research	2.06 ± 1.06
CSS.10: Symptom research interrupts social activities	1.95 ± 1.05
CSS.11: Suggest online-found diagnostic procedures	1.91 ± 1.03
CSS.12: Online research prompts specialist consultation	2.03 ± 1.05
Alexithymia (TAS-20: 20 to 100)	53.95 ± 10.78
TAS.D1: Difficulty identifying feelings	17.40 ± 6.96
TAS.D2: Difficulty describing feelings	13.30 ± 3.37
TAS.D3: Externally oriented thinking	23.25 ± 2.21

^*^SD, standard deviation. FCV-19S, the Fear of COVID-19 Scale; CSS-12, the Short-Form Version of the Cyberchondria Severity Scale; TAS-20, the Toronto 20-item Alexithymia Scale.

### 3.2 Network structure

**Figure 1:**
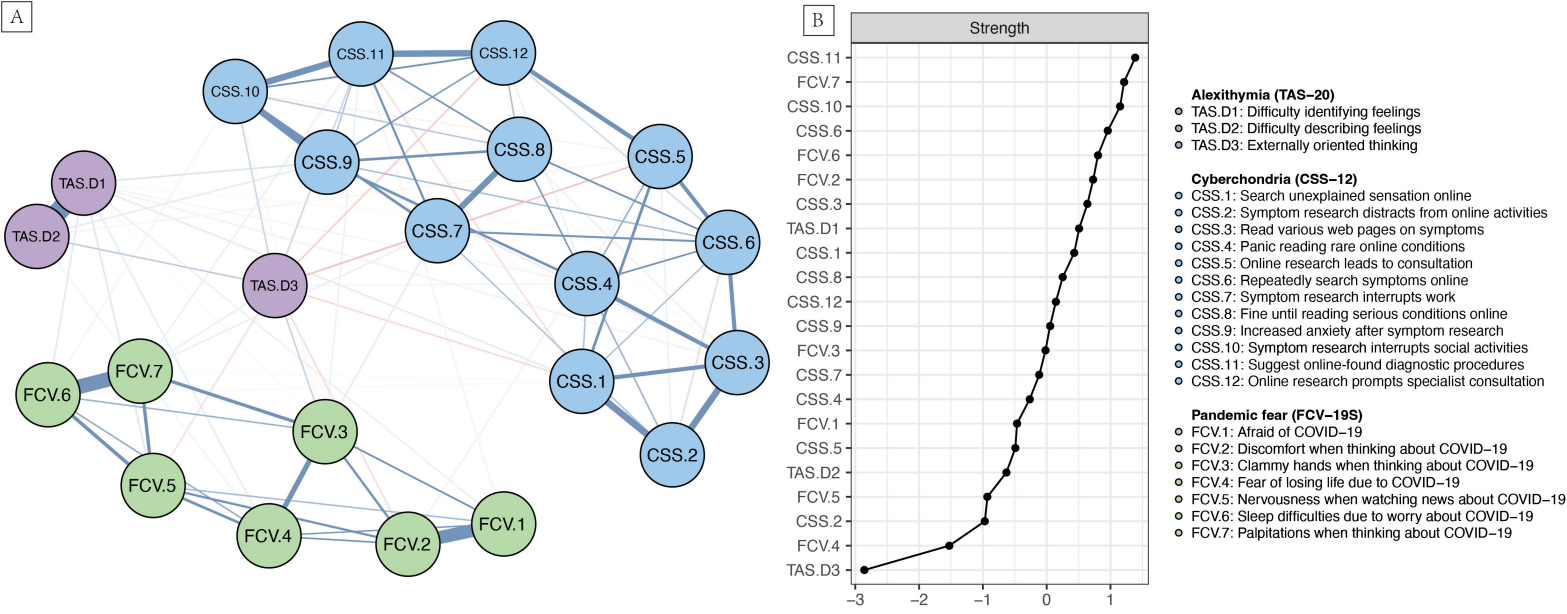
**(A)** The overall network of pandemic fear, cyberchondria, and alexithymia (n = 3977); **(B)** The strength centrality index for the 22 nodes in the network (Z-scored values are presented for each node, with a higher value indicating greater centrality).

**Figure 1A** illustrates the overall network with a density of 0.567. The network encompassed 22 nodes and 131 non-zero edges (mean edge weight: 0.044), predominantly positive. Strong intra-construct partial correlations were observed, particularly within the pandemic fear item cluster (FCV.1-FCV.2: *r* = 0.567; FCV.6-FCV.7: *r* = 0.627) and alexithymia dimensions (TAS.D1-TAS.D2: *r* = 0.800). Within the cyberchondria item cluster, moderate associations were exhibited among several nodes. Cross-construct partial associations, while generally weaker, were also evident. The externally oriented thinking dimension of alexithymia (TAS.D3) displayed a unique pattern, showing negative edges with several FCV and CSS nodes. Comprehensive edge weight details are provided **Supplementary Table S2**.

### 3.3 Node strength centrality and bridge centrality

**Figure 1B** presents the strength centrality index for the 22 nodes in the network (see **Supplementary Table S3** for detailed values). Strength centrality analysis identified CSS.11 (*r*_s_ = 1.388) and FCV.7 (*r*_s_ = 1.212) as the highest-ranking nodes, closely followed by CSS.10 (*r*_s_ = 1.151). In contrast, TAS.D3 exhibited the lowest strength centrality (*r*_s_ = -2.858).

**Figure 2:**
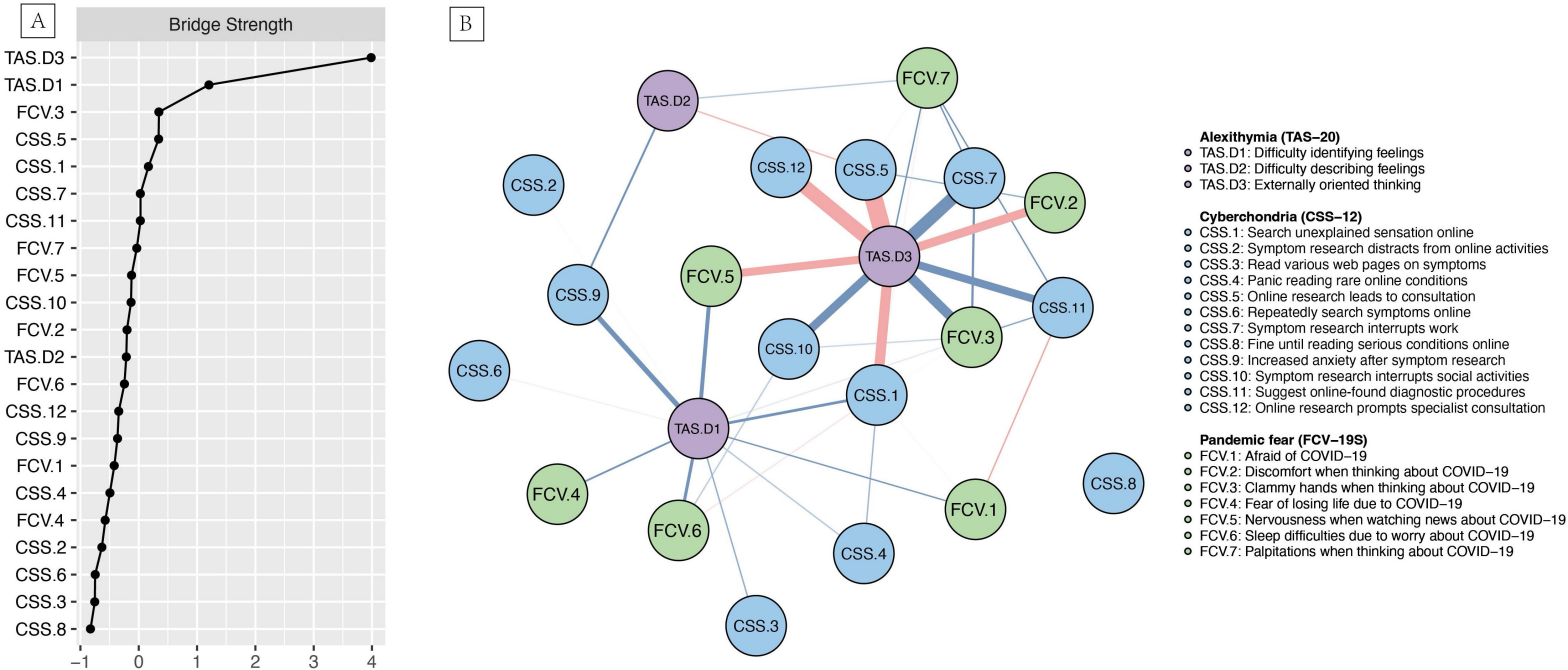
**(A)** Bridge strength centrality index for the psychological network (Z-scored values are presented for each node, with a higher value indicating greater bridge strength); **(B)** Inter-construct connections among pandemic fear, cyberchondria, and alexithymia (n = 3977).

Bridge strength centrality analysis, depicted in **Figure 2A** (See **Supplementary Table S3** for detailed values), revealed that TAS.D3 had the highest bridge strength centrality (*r*_bs_ = 3.986), followed by TAS.D1 (*r*_bs_ = 1.204). A simplified network diagram retaining only inter-construct connections, shown in **Figure 2B**, further elucidates how TAS.D3 and TAS.D1 functioned as key bridge nodes. This visualization demonstrates that these alexithymia dimensions primarily served to connect the pandemic fear and cyberchondria constructs.

### 3.4 Accuracy and stability of the network

**Figure 3:**
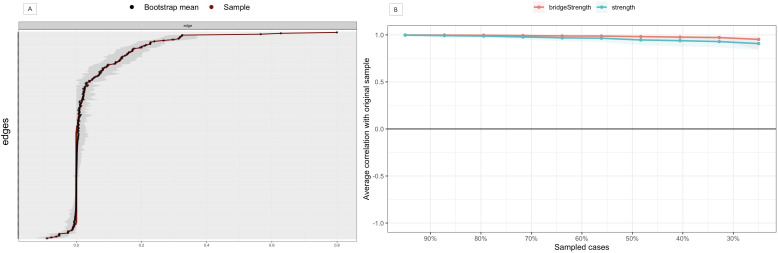
Accuracy and stability analysis of the psychological network. **(A)** Accuracy analysis of the edge weights; **(B)** Stability analysis of the centrality indices.

The bootstrap analysis of edge weights (**Figure 3A**) revealed narrow 95% CIs, indicating high accuracy in the estimated psychological network structure. The case-dropping subset bootstrap procedure (**Figure 3B**) showed that both strength and bridge strength centrality indices had high stability, with CS-coefficients of 0.75 (46). **Supplementary Figures S2–S4** presents the results of the bootstrapped difference tests. The edge weight difference test (**Supplementary Figure S2**) unveiled a large number of statistically significant differences among edges, as evidenced by the predominance of dark boxes. Strong intra-construct edges, particularly within CSS and FCV (e.g., TAS.D1-TAS.D2, FCV.6-FCV.7, and FCV.1-FCV.2), were significantly different from most other edge weights. TAS.D3 exhibited a unique pattern of connections, significantly different from both other TAS dimensions and items from CSS and FCV. The node strength centrality difference test (**Supplementary Figure S3**) confirmed significantly higher centrality for CSS.11, FCV.7, and CSS.10 compared to most other variables, while TAS.D3 exhibited significantly lower centrality. Additionally, the node bridge strength centrality difference test (**Supplementary Figure S4**) verified that TAS.D3 and TAS.D1 were significantly distinct from other nodes in their capacity to connect different constructs within the network.

### 3.5 Subgroup network comparisons by educational level and working experience

Network comparisons were conducted to examine potential differences in network structures stratified by educational level and working experience. The analysis of educational subgroups (junior college or below vs. undergraduate or above) revealed no statistically significant differences in global strength (difference = 0.243, *P* = 0.366) or network structure (*P* = 0.832), as illustrated in **Supplementary Figures S5, S6**. Similarly, comparisons across four working experience subgroups (1-5 vs. 6-10 vs. 11-15 vs. ≥16 years), involving six pairwise analyses, yielded no statistically significant differences in global strength or network structure (all *P* > 0.05), as depicted in **Supplementary Figures S7, S8**. Furthermore, individual edge strength comparisons adjusted for multiple testing showed no significant differences between subgroups in either stratification.

## 4 Discussion

This multi-center network analysis elucidates the complex relationships among pandemic fear, cyberchondria, and alexithymia in nurses following China’s exit from the zero-COVID policy. Our findings reveal a dense network characterized by predominantly positive connections, underscoring the interdependence among these psychological and behavioral phenomena. Strong intra-construct correlations were identified, particularly within the pandemic fear cluster and alexithymia dimensions, along with significant cross-construct associations. Specific components of cyberchondria (CSS.11 and CSS.10) and pandemic fear (FCV.7) demonstrated the highest strength centrality, indicating their pivotal roles in the overall network structure. The externally oriented thinking dimension of alexithymia (TAS.D3) emerged as a critical bridge node, exhibiting the highest bridge strength centrality, followed by difficulty identifying feelings (TAS.D1) (52). These results highlight alexithymia’s significant function in connecting pandemic fear and cyberchondria (52). Additionally, the network demonstrated high accuracy and stability, and remained consistent across different demographic subgroups, which suggests the pervasive nature of these psychological phenomena among the nursing population (54).

The strong intra-construct correlation between general virus-related fear (FCV.1) and cognitive discomfort (FCV.2) within the pandemic fear cluster delineates a close link between these two psychological responses to COVID-19-related fear (9, 11). Similarly, the correlation between sleep disturbances (FCV.6) and physiological arousal (FCV.7) elucidates the interconnectedness of somatic manifestations (9, 11), suggesting underlying self-reinforcing cycles within psychological distress and physiological symptoms, respectively. Within the alexithymia dimensions, a particularly robust correlation exists between difficulty identifying feelings (TAS.D1) and difficulty describing feelings (TAS.D2). This association suggests a generalized difficulty in emotional processing and expression among nurses, where challenges in emotion identification are inextricably linked to impairments in articulation (29, 56). These findings underscore the internal coherence of the pandemic fear and alexithymia constructs (11, 26, 39) and indicate that interventions targeting one aspect could potentially influence related components.

Node centrality analyses revealed that CSS.11, FCV.7, and CSS.10 exhibited the highest strength centrality, indicating their critical influence on the overall network structure (49). The prominence of CSS.11 (“Suggest online-found diagnostic procedures”), aligning with previous research on the challenges healthcare professionals face in the era of accessible online health information, may reflect an interplay between professional knowledge, personal health anxiety, and the influence of online information-seeking behaviors (16, 57). The high centrality of CSS.10 (“Symptom research interrupts social activities”) suggests that compulsive behaviors driven by health anxiety significantly impacts personal lives and leisure time, potentially precipitating social loneliness and isolation (58). Additionally, the high centrality of FCV.7 (“Palpitations when thinking about COVID-19”) highlights the psychophysiological manifestations of COVID-19 fear, indicating a strong physiological component to pandemic-related fear among nurses (11, 14). This finding suggests that nurses’ maladaptive responses encompass substantial somatic reactions, potentially compromising their overall well-being, work performance, and interpersonal dynamics.

In addition, TAS.D3 demonstrated significant negative associations with several other nodes and presented low strength centrality coupled with the highest bridge strength. This implies a potential protective mechanism, wherein externally oriented thinking might attenuate other psychological symptoms. Consequently, nurses with a propensity for externally oriented thinking may exhibit reduced vulnerability to pandemic-related fear and compulsive health-related behaviors. Furthermore, this finding suggests that while externally oriented thinking may not be central to the network’s overall intensity, it serves as a crucial bridge connecting diverse psychological and behavioral constructs (52). Therefore, nursing managers could consider incorporating strategies that promote objective fact-focused and problem-solving approaches in staff support programs, while also cultivating skills in emotional awareness and expression (26, 29). Concurrently, TAS.D1 (difficulty identifying feelings) exhibited significant positive associations with other nodes and the second-highest bridge strength in the network, suggesting that enhancing nurses’ emotional identification competencies might be an effective intervention target in fostering adaptive psychological and behavioral responses. Healthcare administrators might consider implementing evidence-based workplace initiatives that focus on augmenting emotional awareness and recognition skills (18, 26, 56). This dual approach, addressing both externally oriented thinking and emotional identification, could potentially yield synergistic effects in promoting psychological well-being and healthier behaviors among nursing professionals.

The robustness and reliability of our network analysis were rigorously assessed through bootstrapping procedures, yielding compelling evidence of the network’s accuracy and stability (46). The bootstrapped difference tests corroborated the network’s structural integrity and extended our earlier findings (46). Moreover, the subgroup network comparison analyses resulted in absence of statistically significant differences in global strength and network structure across educational levels and working experience categories, suggesting a remarkable consistency in the interrelationships among pandemic fear, cyberchondria, and alexithymia (46, 54). This homogeneity in network structure suggests that the psychological dynamics underlying these constructs may be relatively invariant to formal education background and professional experience. Such consistency implies that unified approaches to addressing pandemic-related psychological distress and maladaptive health behaviors might be effective across the entire nursing workforce. Interventions should, therefore, focus on shared experiences and challenges of the nursing profession during the uncertain transition period, targeting the intersection of psychological factors and health behaviors, while stratified approaches based on education or experience may not be essential.

## 5 Limitations

This study has several limitations. Firstly, the cross-sectional nature of our data limits causal inferences and the interpretation of temporal dynamics. Moreover, the timing of data collection—conducted immediately after China’s exit from the zero-COVID policy—may restrict the applicability of our findings to periods beyond the immediate aftermath of the policy shift. Since network structures may evolve over time, especially during such transitions, longitudinal studies are necessary to capture these dynamics more comprehensively. Secondly, our sample predominantly comprised participants from the western and central regions of mainland China, potentially limiting the generalizability of our findings. Cultural and regional variations in healthcare systems and pandemic experiences could influence the observed network structures. Thirdly, despite our large sample size, certain demographic groups were underrepresented, including males, those with senior professional titles, uninfected individuals, and those at the extremes of socioeconomic status, preventing comprehensive network comparisons across these subgroups. Fourthly, reliance on self-report measures collected through voluntary online questionnaires may have introduced reporting bias and selection bias, potentially affecting the representativeness of our sample. Additionally, our decision to combine item-level data for pandemic fear and cyberchondria with dimension-level data for alexithymia in the network analysis, while aiming to balance detail and interpretability, may have introduced methodological bias. This mixed-level approach potentially overrepresented alexithymia’s influence while obscuring its fine-grained internal structure, and may have contributed to the attenuated conditioned edge weights observed between alexithymia and the other two constructs in the network. Future research could employ longitudinal designs with multi-regional, demographically diverse samples, incorporating both self-report and objective measures to enhance network robustness and generalizability.

## 6 Conclusion

The study unravels the complex relationships among pandemic fear, cyberchondria, and alexithymia in Chinese nurses following the cessation of the zero-COVID policy, providing insights into nurses’ psychological states during a critical transition. Our network analysis has identified specific aspects of cyberchondria and pandemic fear as exhibiting the highest strength centrality within the psychological and behavioral network. Notably, the externally oriented thinking dimension of alexithymia emerged as a crucial bridge node, mediating the connection between pandemic fear and cyberchondria. These findings underscore the imperative for targeted interventions focusing on key network components, particularly balancing externally oriented thinking with emotional awareness, to disrupt the vicious cycle of maladaptive pandemic fear responses and dysfunctional online health information-seeking behaviors. Healthcare organizations should prioritize support services that promote balanced, objective fact-focused and problem-solving approaches while concurrently fostering skills in emotional awareness and recognition, thereby mitigating the risk of maladaptive psychological responses and promoting healthier behaviors among nursing professionals. Future research should explore the causal mechanisms underlying these relationships through longitudinal studies and evaluate the effectiveness of interventions targeting these key network components, further elucidating the dynamic interplay between psychology and health behaviors in healthcare professionals navigating unprecedented challenges.

## Data Availability

The original contributions presented in the study are included in the article/**Supplementary Material**. Further inquiries can be directed to the corresponding authors.
