# Supplementary material for: Unraveling the relationships among pandemic fear, cyberchondria, and alexithymia after China’s exit from the zero-COVID policy: insights from a multi-center network analysis
Source: Front Psychiatry. 2024 Nov 14;15:1489961. doi: 10.3389/fpsyt.2024.1489961 (PMC11602484; doi:10.3389/fpsyt.2024.1489961)
Supplement: Supplementary file 1 [file DataSheet1.pdf]

Supplementary Materials

Content

Table S1. Pearson correlation matrix of pandemic fear (FCV-19S), cyberchondria (CSS-12), and alexithymia (TAS-20) (N = 3977). 2

Table S2. GLASSO-estimated edge weight matrix for the network of pandemic fear (FCV-19S), cyberchondria (CSS-12), and alexithymia (TAS-20) (N = 3977).......... 3

Table S3. Standardized values for nodes' strength, closeness, betweenness, and bridge strength centralities. 4

Figure S1. Heatmap of the Pearson correlations among pandemic fear (FCV-19S), cyberchondria (CSS-12), and alexithymia (TAS-20). 5

Figure S2. Bootstrapped difference test of edge weights. 6

Figure S3. Bootstrapped difference test of the node strength centrality. 7

Figure S4. Bootstrapped difference test of the node bridge strength centrality 7

Figure S5. Educational level-stratified psychological networks of pandemic fear, cyberchondria, and alexithymia. (A) Junior college or below (n = 1761); (B) Undergraduate or above (n = 2216). 8

Figure S6. Strength, closeness, and betweenness centrality indices for the psychological networks stratified by educational level. 8

Figure S7. Working experience-stratified psychological networks of pandemic fear, cyberchondria, and alexithymia. (A) 1-5 years group (n = 980); (B) 6-10 years group (n = 1033); (C) 11-15 years group (n = 1128); (D) ≥ 16 years group (n = 836). 9

Figure S8. Strength, closeness, and betweenness centrality indices for the psychological networks stratified by working experience. 10

Table S1. Pearson correlation matrix of pandemic fear (FCV-19S), cyberchondria (CSS-12), and alexithymia (TAS-20) (N = 3977).

Variables	CSS.1	CSS.2	CSS.3	CSS.4	CSS.5	CSS.6	CSS.7	CSS.8	CSS.9	CSS.10	CSS.11	CSS.12	CSS	FCV.1	FCV.2	FCV.3	FCV.4	FCV.5	FCV.6	FCV.7	FCV	TAS.D1	TAS.D2	TAS.D3	TAS
**CSS.1**	1.000	0.800	0.802	0.744	0.707	0.752	0.641	0.660	0.669	0.616	0.594	0.642	0.816	0.368	0.381	0.373	0.376	0.396	0.380	0.380	0.419	0.492	0.442	0.044	0.465
**CSS.2**	0.800	1.000	0.838	0.775	0.690	0.775	0.716	0.720	0.710	0.685	0.667	0.672	0.855	0.392	0.406	0.432	0.418	0.439	0.452	0.450	0.471	0.520	0.479	0.117	0.510
**CSS.3**	0.802	0.838	1.000	0.832	0.722	0.839	0.750	0.770	0.762	0.725	0.714	0.723	0.895	0.401	0.419	0.441	0.425	0.453	0.464	0.459	0.483	0.549	0.504	0.126	0.538
**CSS.4**	0.744	0.775	0.832	1.000	0.738	0.832	0.768	0.807	0.808	0.763	0.748	0.758	0.904	0.426	0.443	0.465	0.464	0.474	0.492	0.487	0.512	0.560	0.509	0.144	0.550
**CSS.5**	0.707	0.690	0.722	0.738	1.000	0.778	0.697	0.703	0.702	0.662	0.665	0.748	0.833	0.385	0.404	0.397	0.408	0.411	0.408	0.405	0.445	0.464	0.408	0.029	0.433
**CSS.6**	0.752	0.775	0.839	0.832	0.778	1.000	0.813	0.830	0.811	0.777	0.759	0.784	0.920	0.419	0.442	0.460	0.451	0.464	0.478	0.474	0.503	0.560	0.518	0.124	0.549
**CSS.7**	0.641	0.716	0.750	0.768	0.697	0.813	1.000	0.861	0.805	0.835	0.827	0.775	0.894	0.433	0.451	0.523	0.480	0.486	0.531	0.537	0.542	0.538	0.509	0.201	0.548
**CSS.8**	0.660	0.720	0.770	0.807	0.703	0.830	0.861	1.000	0.850	0.840	0.837	0.814	0.913	0.432	0.449	0.504	0.481	0.484	0.515	0.513	0.532	0.556	0.522	0.176	0.558
**CSS.9**	0.669	0.710	0.762	0.808	0.702	0.811	0.805	0.850	1.000	0.871	0.832	0.821	0.909	0.435	0.458	0.487	0.479	0.494	0.509	0.503	0.530	0.592	0.554	0.162	0.589
**CSS.10**	0.616	0.685	0.725	0.763	0.662	0.777	0.835	0.840	0.871	1.000	0.875	0.831	0.893	0.439	0.461	0.523	0.484	0.504	0.536	0.539	0.549	0.573	0.537	0.202	0.579
**CSS.11**	0.594	0.667	0.714	0.748	0.665	0.759	0.827	0.837	0.832	0.875	1.000	0.850	0.882	0.415	0.443	0.513	0.469	0.480	0.524	0.530	0.531	0.539	0.510	0.195	0.547
**CSS.12**	0.642	0.672	0.723	0.758	0.748	0.784	0.775	0.814	0.821	0.831	0.850	1.000	0.887	0.412	0.434	0.469	0.454	0.466	0.482	0.483	0.504	0.532	0.488	0.101	0.517
**CSS**	0.816	0.855	0.895	0.904	0.833	0.920	0.894	0.913	0.909	0.893	0.882	0.887	1.000	0.468	0.489	0.526	0.508	0.523	0.543	0.543	0.568	0.611	0.564	0.152	0.602
**FCV.1**	0.368	0.392	0.401	0.426	0.385	0.419	0.433	0.432	0.435	0.439	0.415	0.412	0.468	1.000	0.874	0.777	0.768	0.753	0.712	0.716	0.886	0.528	0.487	0.086	0.511
**FCV.2**	0.381	0.406	0.419	0.443	0.404	0.442	0.451	0.449	0.458	0.461	0.443	0.434	0.489	0.874	1.000	0.793	0.783	0.777	0.727	0.726	0.899	0.537	0.499	0.072	0.517
**FCV.3**	0.373	0.432	0.441	0.465	0.397	0.460	0.523	0.504	0.487	0.523	0.513	0.469	0.526	0.777	0.793	1.000	0.814	0.775	0.812	0.823	0.913	0.565	0.527	0.167	0.564
**FCV.4**	0.376	0.418	0.425	0.464	0.408	0.451	0.480	0.481	0.479	0.484	0.469	0.454	0.508	0.768	0.783	0.814	1.000	0.789	0.783	0.776	0.902	0.556	0.514	0.121	0.544
**FCV.5**	0.396	0.439	0.453	0.474	0.411	0.464	0.486	0.484	0.494	0.504	0.480	0.466	0.523	0.753	0.777	0.775	0.789	1.000	0.839	0.838	0.911	0.572	0.532	0.095	0.555
**FCV.6**	0.380	0.452	0.464	0.492	0.408	0.478	0.531	0.515	0.509	0.536	0.524	0.482	0.543	0.712	0.727	0.812	0.783	0.839	1.000	0.924	0.912	0.587	0.548	0.160	0.584
**FCV.7**	0.380	0.450	0.459	0.487	0.405	0.474	0.537	0.513	0.503	0.539	0.530	0.483	0.543	0.716	0.726	0.823	0.776	0.838	0.924	1.000	0.913	0.582	0.547	0.167	0.581
**FCV**	0.419	0.471	0.483	0.512	0.445	0.503	0.542	0.532	0.530	0.549	0.531	0.504	0.568	0.886	0.899	0.913	0.902	0.911	0.912	0.913	1.000	0.619	0.576	0.136	0.608
**TAS.D1**	0.492	0.520	0.549	0.560	0.464	0.560	0.538	0.556	0.592	0.573	0.539	0.532	0.611	0.528	0.537	0.565	0.556	0.572	0.587	0.582	0.619	1.000	0.899	0.200	0.968
**TAS.D2**	0.442	0.479	0.504	0.509	0.408	0.518	0.509	0.522	0.554	0.537	0.510	0.488	0.564	0.487	0.499	0.527	0.514	0.532	0.548	0.547	0.576	0.899	1.000	0.213	0.937
**TAS.D3**	0.044	0.117	0.126	0.144	0.029	0.124	0.201	0.176	0.162	0.202	0.195	0.101	0.152	0.086	0.072	0.167	0.121	0.095	0.160	0.167	0.136	0.200	0.213	1.000	0.400
**TAS**	0.465	0.510	0.538	0.550	0.433	0.549	0.548	0.558	0.589	0.579	0.547	0.517	0.602	0.511	0.517	0.564	0.544	0.555	0.584	0.581	0.608	0.968	0.937	0.400	1.000

Note. Grey-shaded areas represent submatrices of intra-construct Pearson correlations.

Table S2. GLASSO-estimated edge weight matrix for the network of pandemic fear (FCV-19S), cyberchondria (CSS-12), and alexithymia (TAS-20) (N = 3977).

Variables	CSS.1	CSS.2	CSS.3	CSS.4	CSS.5	CSS.6	CSS.7	CSS.8	CSS.9	CSS.10	CSS.11	CSS.12	FCV.1	FCV.2	FCV.3	FCV.4	FCV.5	FCV.6	FCV.7	TAS.D1	TAS.D2	TAS.D3
**CSS.1**	0.000	**0.324**	**0.229**	0.075	0.179	0.072	-0.001	0.000	0.000	0.000	-0.054	0.000	0.010	0.004	-0.010	0.000	0.001	-0.014	-0.011	0.031	0.000	-0.060
**CSS.2**	**0.324**	0.000	**0.320**	0.092	0.022	0.042	0.075	0.000	0.000	0.006	0.000	0.000	0.000	0.000	0.000	0.000	0.008	0.009	0.000	0.010	0.001	0.010
**CSS.3**	**0.229**	**0.320**	0.000	**0.227**	0.000	**0.228**	0.012	0.023	0.026	0.000	0.013	0.000	0.000	0.000	0.000	0.000	0.000	0.000	0.000	0.021	0.000	0.001
**CSS.4**	0.075	0.092	**0.227**	0.000	0.098	0.136	0.000	0.126	0.165	0.000	0.000	0.023	0.000	0.000	0.000	0.007	0.006	0.005	0.000	0.018	0.000	0.020
**CSS.5**	0.179	0.022	0.000	0.098	0.000	**0.215**	0.030	0.000	0.000	-0.025	0.000	**0.239**	0.006	0.021	-0.006	0.007	0.000	0.000	-0.010	0.000	-0.022	-0.091
**CSS.6**	0.072	0.042	**0.228**	0.136	**0.215**	0.000	0.149	0.138	0.082	0.000	0.000	0.048	0.000	0.000	-0.006	0.000	0.000	0.000	0.000	0.011	0.005	0.000
**CSS.7**	-0.001	0.075	0.012	0.000	0.030	0.149	0.000	**0.298**	0.000	0.171	0.154	0.000	0.000	0.000	0.028	0.000	0.000	0.000	0.025	-0.001	0.000	0.069
**CSS.8**	0.000	0.000	0.023	0.126	0.000	0.138	**0.298**	0.000	0.173	0.070	0.134	0.081	0.000	0.000	0.000	0.005	0.000	0.000	0.000	0.000	0.000	0.008
**CSS.9**	0.000	0.000	0.026	0.165	0.000	0.082	0.000	0.173	0.000	**0.324**	0.072	0.119	0.000	0.000	-0.006	0.000	0.000	0.000	0.000	0.040	0.028	0.000
**CSS.10**	0.000	0.006	0.000	0.000	-0.025	0.000	0.171	0.070	**0.324**	0.000	**0.314**	0.142	0.000	0.000	0.015	0.000	0.000	0.017	0.002	0.009	0.007	0.053
**CSS.11**	-0.054	0.000	0.013	0.000	0.000	0.000	0.154	0.134	0.072	**0.314**	0.000	**0.317**	-0.025	0.000	0.022	0.000	0.000	0.001	0.025	0.000	0.000	0.050
**CSS.12**	0.000	0.000	0.000	0.023	**0.239**	0.048	0.000	0.081	0.119	0.142	**0.317**	0.000	0.000	0.000	0.000	0.000	0.000	0.000	0.000	0.000	0.000	-0.076
**FCV.1**	0.010	0.000	0.000	0.000	0.006	0.000	0.000	0.000	0.000	0.000	-0.025	0.000	0.000	**0.567**	0.139	0.121	0.084	0.000	0.000	0.024	0.000	0.000
**FCV.2**	0.004	0.000	0.000	0.000	0.021	0.000	0.000	0.000	0.000	0.000	0.000	0.000	**0.567**	0.000	0.160	0.133	0.156	0.000	0.000	0.004	0.009	-0.054
**FCV.3**	-0.010	0.000	0.000	0.000	-0.006	-0.006	0.028	0.000	-0.006	0.015	0.022	0.000	0.139	0.160	0.000	**0.267**	0.000	0.094	**0.201**	0.013	0.000	0.058
**FCV.4**	0.000	0.000	0.000	0.007	0.007	0.000	0.000	0.005	0.000	0.000	0.000	0.000	0.121	0.133	**0.267**	0.000	0.173	0.101	0.018	0.027	0.000	0.000
**FCV.5**	0.001	0.008	0.000	0.006	0.000	0.000	0.000	0.000	0.000	0.000	0.000	0.000	0.084	0.156	0.000	0.173	0.000	**0.207**	**0.201**	0.035	0.002	-0.051
**FCV.6**	-0.014	0.009	0.000	0.005	0.000	0.000	0.000	0.000	0.000	0.017	0.001	0.000	0.000	0.000	0.094	0.101	**0.207**	0.000	**0.627**	0.032	0.002	0.009
**FCV.7**	-0.011	0.000	0.000	0.000	-0.010	0.000	0.025	0.000	0.000	0.002	0.025	0.000	0.000	0.000	**0.201**	0.018	**0.201**	**0.627**	0.000	0.000	0.017	0.025
**TAS.D1**	0.031	0.010	0.021	0.018	0.000	0.011	-0.001	0.000	0.040	0.009	0.000	0.000	0.024	0.004	0.013	0.027	0.035	0.032	0.000	0.000	**0.800**	0.008
**TAS.D2**	0.000	0.001	0.000	0.000	-0.022	0.005	0.000	0.000	0.028	0.007	0.000	0.000	0.000	0.009	0.000	0.000	0.002	0.002	0.017	**0.800**	0.000	0.066
**TAS.D3**	-0.060	0.010	0.001	0.020	-0.091	0.000	0.069	0.008	0.000	0.053	0.050	-0.076	0.000	-0.054	0.058	0.000	-0.051	0.009	0.025	0.008	0.066	0.000

Note. Bold values indicate partial correlation coefficients greater than 0.3; Grey-shaded areas represent submatrices of intra-construct partial correlations.

Table S3. Standardized values for nodes' strength, closeness, betweenness, and bridge strength centralities.

Nodes	Strength values	Nodes	Bridge strength values	Nodes	Closeness values	Nodes	Betweenness values
**CSS.11**	**1.388**	**TAS.D3**	**3.986**	CSS.5	1.760	**TAS.D3**	**3.650**
**FCV.7**	**1.212**	**TAS.D1**	**1.204**	**TAS.D3**	**1.746**	CSS.5	1.649
**CSS.10**	**1.151**	FCV.3	0.347	CSS.12	1.215	FCV.3	1.095
CSS.6	0.957	CSS.5	0.34	CSS.6	0.965	CSS.12	0.372
FCV.6	0.804	CSS.1	0.168	CSS.7	0.964	CSS.6	0.116
FCV.2	0.730	CSS.11	0.03	CSS.11	0.676	FCV.2	0.031
CSS.3	0.638	CSS.7	0.03	CSS.8	0.470	FCV.7	-0.097
TAS.D1	0.509	FCV.7	-0.033	CSS.1	0.455	CSS.1	-0.139
CSS.1	0.432	FCV.5	-0.123	CSS.10	0.432	CSS.9	-0.139
CSS.8	0.251	CSS.10	-0.132	CSS.9	0.253	CSS.3	-0.267
CSS.12	0.144	FCV.2	-0.202	CSS.4	0.183	CSS.7	-0.310
CSS.9	0.053	TAS.D2	-0.213	CSS.3	0.178	TAS.D1	-0.352
FCV.3	-0.018	FCV.6	-0.246	CSS.2	0.020	TAS.D2	-0.352
CSS.7	-0.115	CSS.12	-0.343	FCV.3	-0.525	CSS.4	-0.437
CSS.4	-0.267	CSS.9	-0.365	FCV.2	-0.756	CSS.11	-0.480
FCV.1	-0.465	FCV.1	-0.418	FCV.5	-0.771	CSS.10	-0.523
CSS.5	-0.491	CSS.4	-0.496	FCV.1	-1.053	FCV.5	-0.523
TAS.D2	-0.632	FCV.4	-0.572	FCV.4	-1.073	FCV.6	-0.608
FCV.5	-0.928	CSS.2	-0.633	FCV.7	-1.181	CSS.8	-0.650
CSS.2	-0.970	CSS.6	-0.748	TAS.D2	-1.296	FCV.4	-0.650
FCV.4	-1.526	CSS.3	-0.753	TAS.D1	-1.315	FCV.1	-0.693
TAS.D3	-2.859	CSS.8	-0.827	FCV.6	-1.346	CSS.2	-0.693

Note. Nodes are arranged in descending order according to their respective centrality values. This study focuses on strength and bridge strength, as closeness and betweenness are often less reliable in psychological networks.

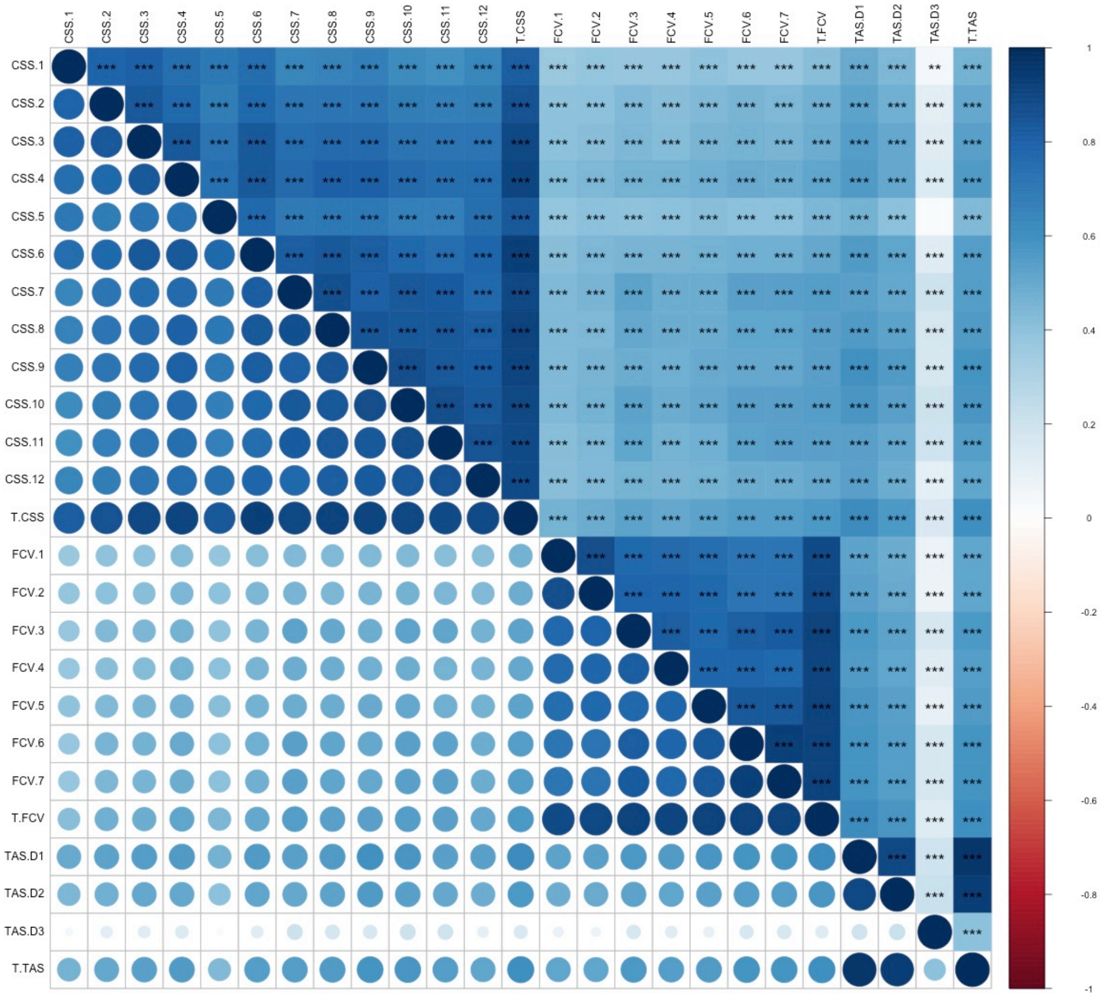

Figure S1. Heatmap of the Pearson correlations among pandemic fear (FCV-19S), cyberchondria (CSS-12), and alexithymia (TAS-20).

Note. * < 0.05, ** < 0.01, *** < 0.001.

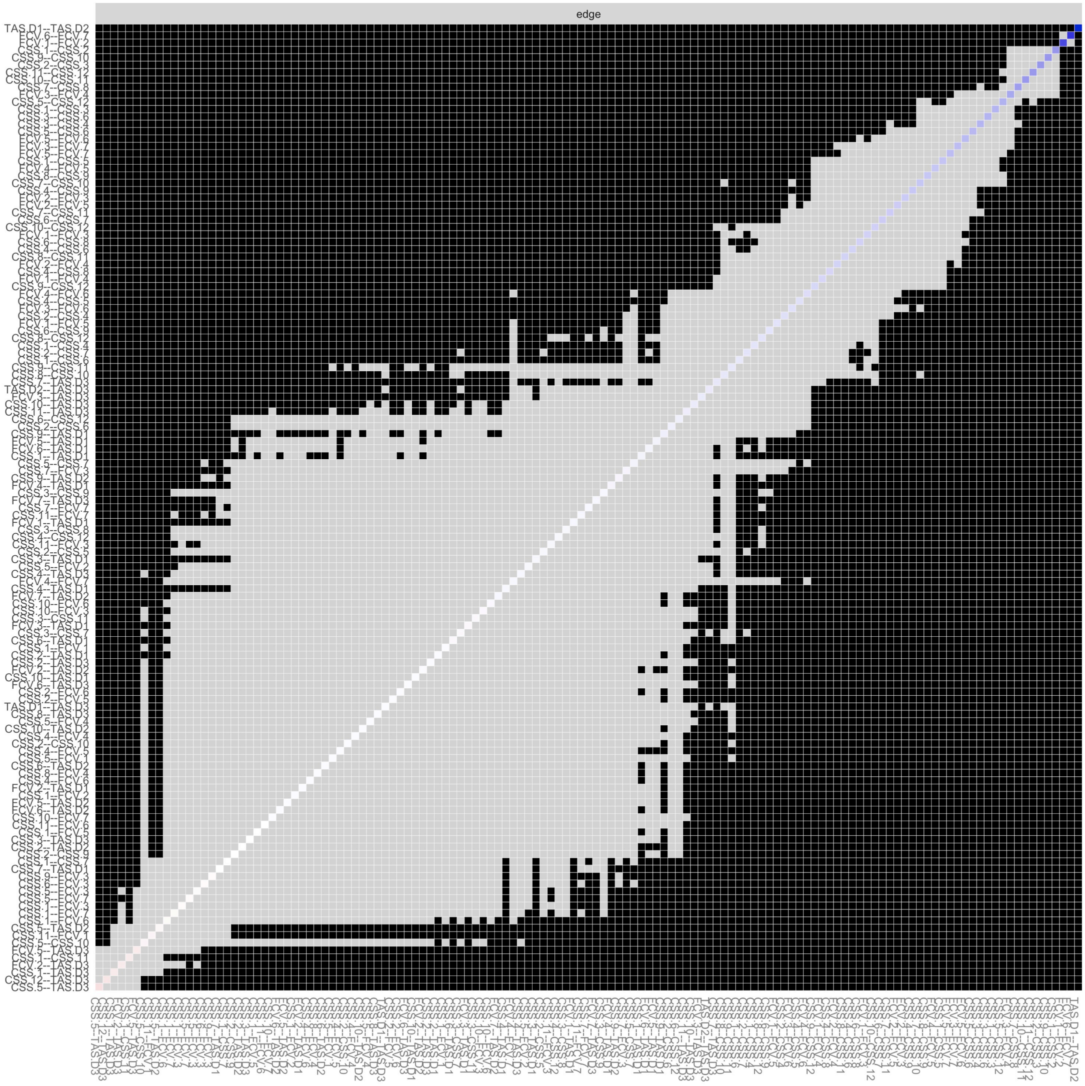

Figure S2. Bootstrapped difference test of edge weights.

Note. The y-axis and x-axis represent individual nodes within the psychological network. Colored boxes on the diagonal indicate the corresponding color of the edge within the network. Gray boxes indicate non-significant differences, while black boxes indicate significant differences.

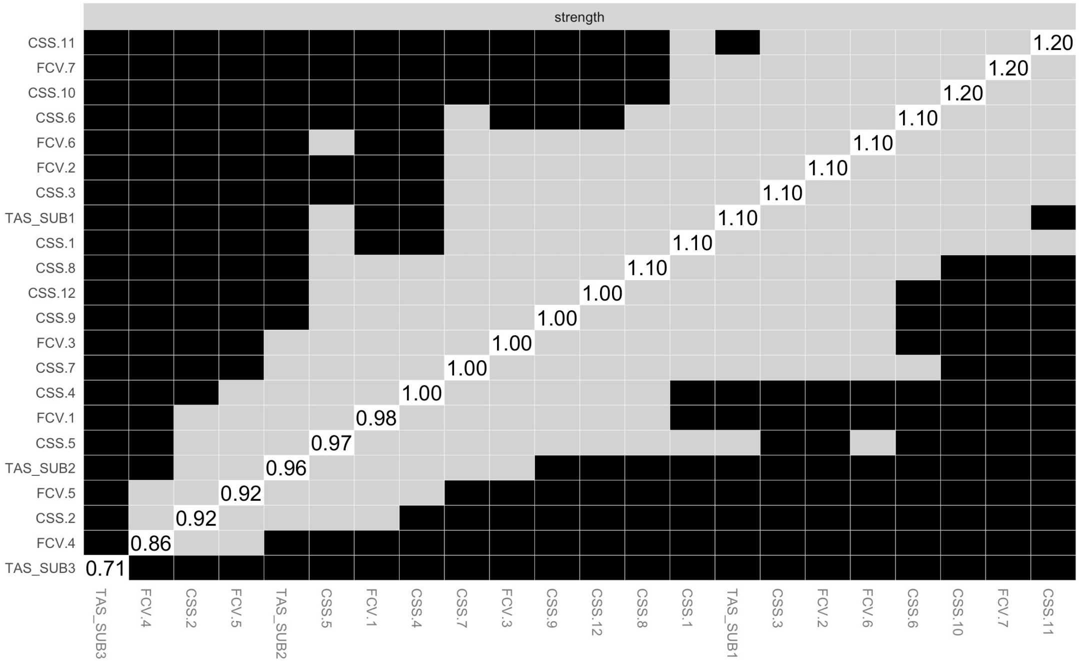

Figure S3. Bootstrapped difference test of the node strength centrality.

Note. The y-axis and x-axis represent individual nodes within the psychological network. Strength centrality values are plotted on the diagonal. Gray boxes indicate non-significant differences, while black boxes indicate significant differences.

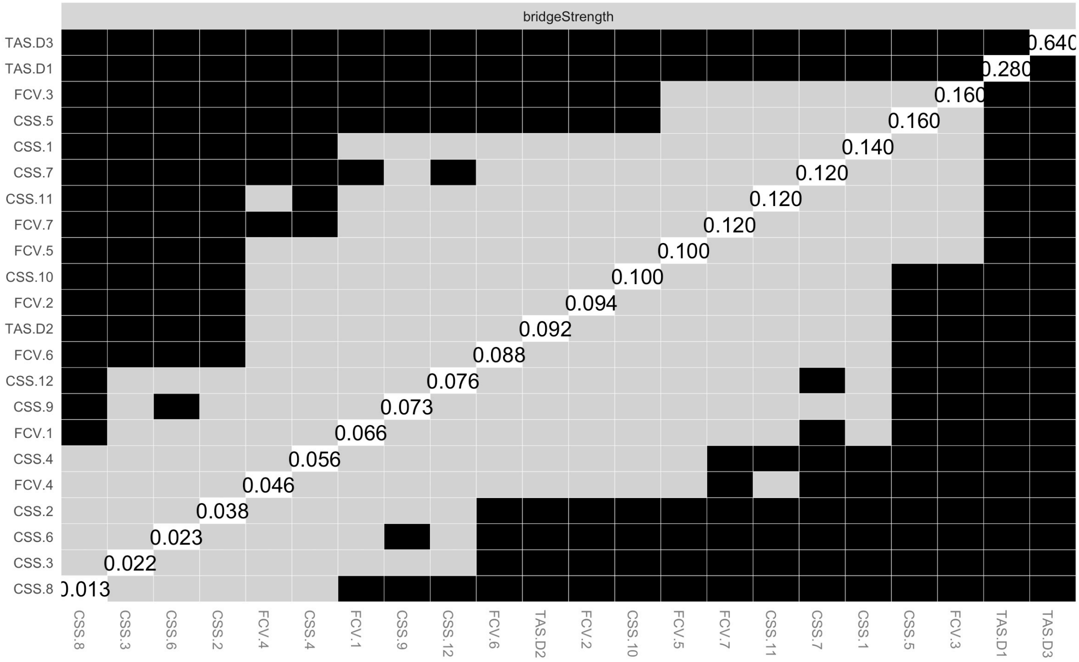

Figure S4. Bootstrapped difference test of the node bridge strength centrality.

Note. The y-axis and x-axis represent individual nodes within the psychological network. Node bridge strength values are plotted on the diagonal. Gray boxes indicate non-significant differences, while black boxes indicate significant differences.

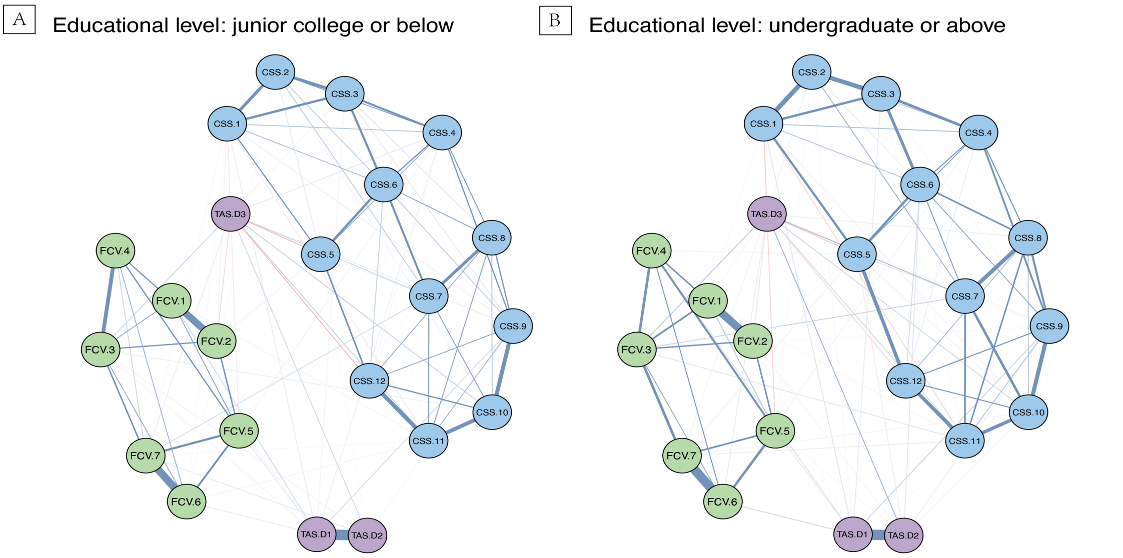

Figure S5. Educational level-stratified psychological networks of pandemic fear, cyberchondria, and alexithymia. (A) Junior college or below (n = 1761); (B) Undergraduate or above (n = 2216).

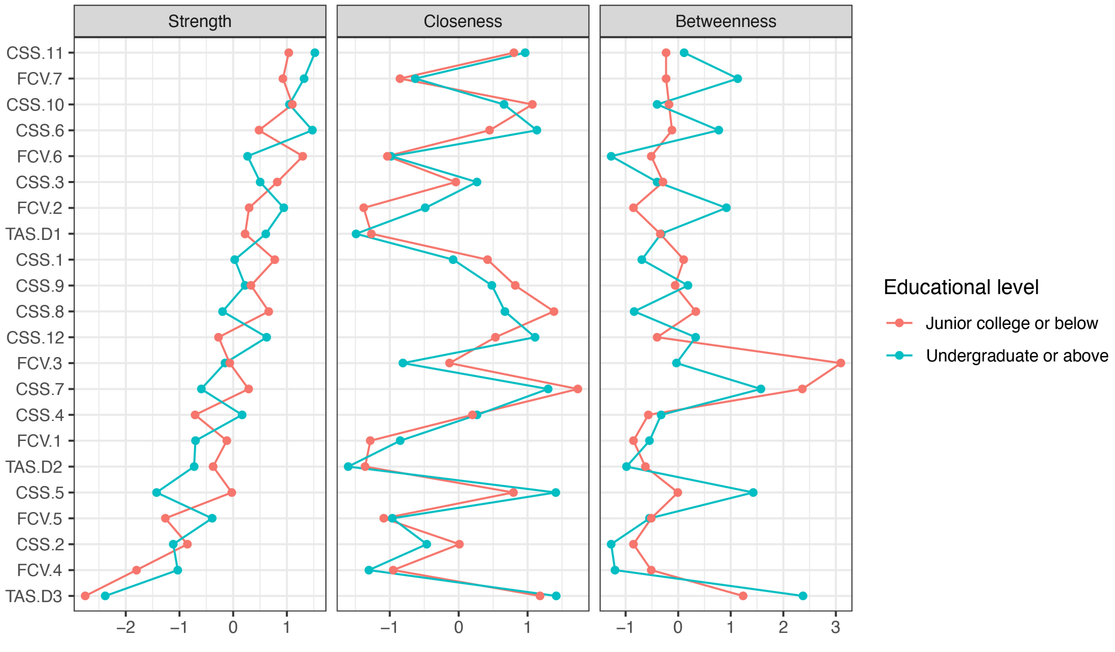

Figure S6. Strength, closeness, and betweenness centrality indices for the psychological networks stratified by educational level.

Note. Z-scored values are presented for each node, with a higher value indicating greater centrality. This study focuses on the node strength index, as closeness and betweenness are often less reliable in psychological networks.

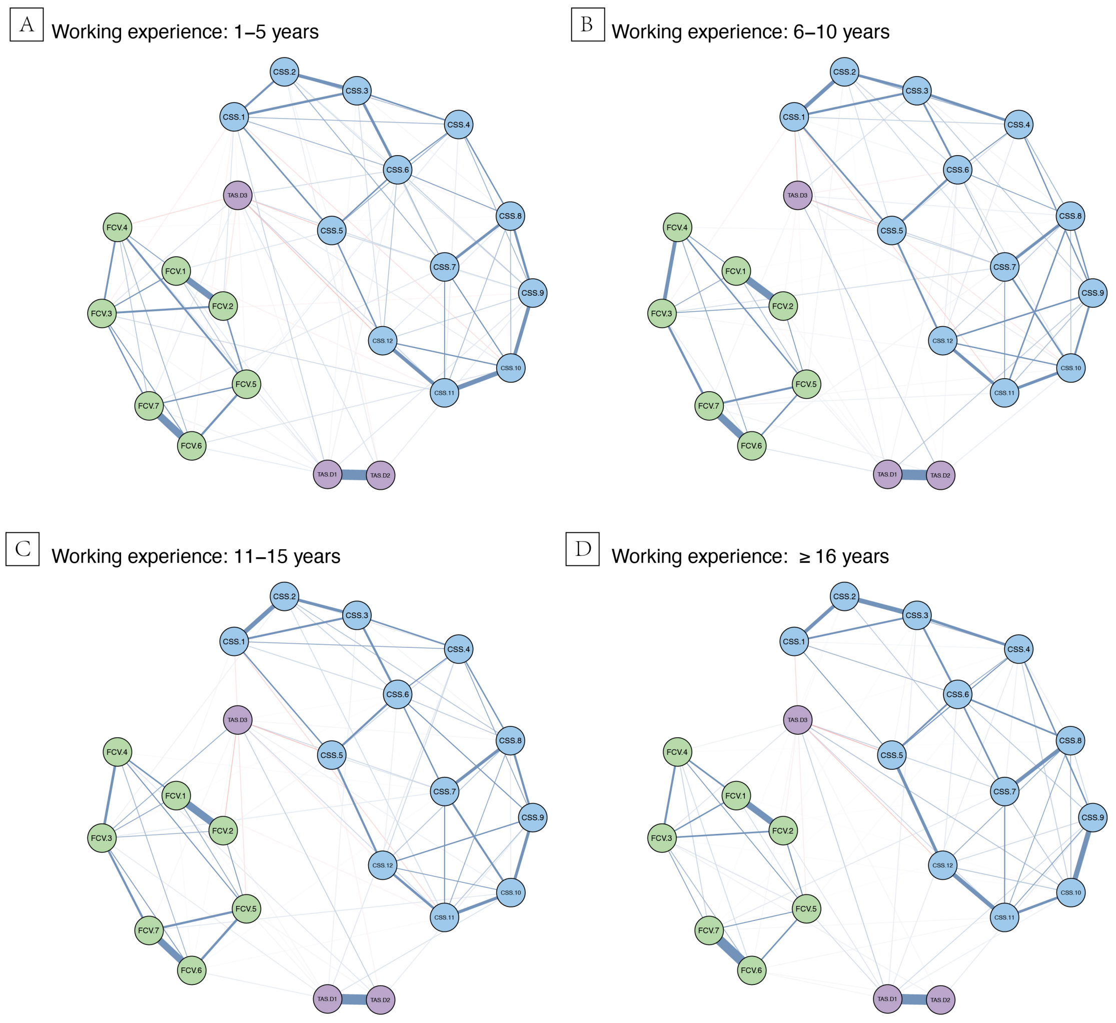

Figure S7. Working experience-stratified psychological networks of pandemic fear, cyberchondria, and alexithymia. (A) 1-5 years group (n = 980); (B) 6-10 years group (n = 1033); (C) 11-15 years group (n = 1128); (D) ≥ 16 years group (n = 836).

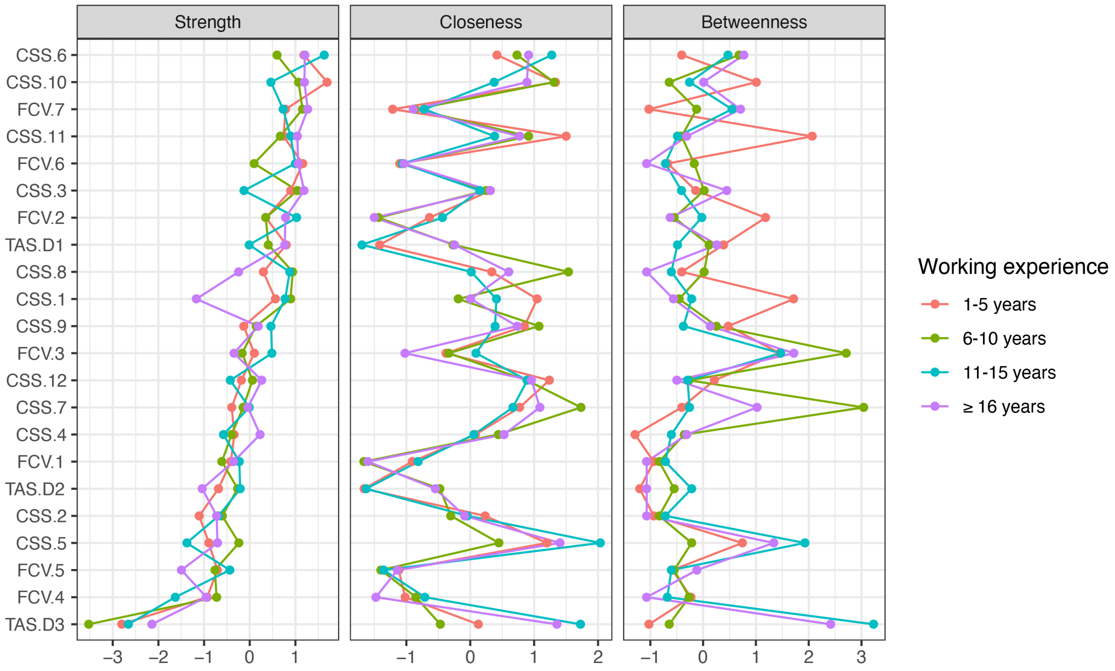

Figure S8. Strength, closeness, and betweenness centrality indices for the psychological networks stratified by working experience.

Note. Z-scored values are presented for each node, with a higher value indicating greater centrality. This study focuses on the node strength index, as closeness and betweenness are often less reliable in psychological networks.
