# Supplementary material for: Unraveling the relationships among pandemic fear, cyberchondria, and alexithymia after China’s exit from the zero-COVID policy: insights from a multi-center network analysis
Source: Front Psychiatry. 2024 Nov 14;15:1489961. doi: 10.3389/fpsyt.2024.1489961 (PMC11602484; doi:10.3389/fpsyt.2024.1489961)
Supplement: Supplementary file 2 [file DataSheet2.pdf]

Supplementary Materials

````
---
output:
  word_document: default
  html_document: default
  pdf_document: default
---

#1.loading pkts
```{r}
library(ggplot2)
library(qgraph)
library(corpcor)
library(ggplot2)
library(bootnet)
library(NetworkComparisonTest)
library(networktools)
library(IsingFit)
library(writexl)
library(corrplot)
```

#2.loading data
```{r}
data <- read.csv("data.csv")
data <- na.omit(data)
col_names <- colnames(data)
selected_cols <- grep("^(CSS|FCV|TAS.D)", col_names, value = TRUE)
selected_cols_cor <- grep("^(CSS|FCV|TAS.D|T.TAS|T.CSS|T.FCV)", col_names, value
= TRUE)
data_selected <- data[, selected_cols]
data_selected_cor <- data[, selected_cols_cor]
set.seed(6666)
````
````

````
```
````

#3.Conduct correlation analysis and plot the hotmap

````
```{r}
cor_data <- data_selected_cor
#Computed correlation matrix
M<-cor(cor_data, method = "pearson")
#Print correlation matrix
print(M)
write.csv(M,file="Table.CorrelationMatrix.csv")
#Significance test of correlation
testRes<-cor.mtest(cor_data,conf.level = 0.95)

par(mfrow=c(2,3))
#Save correlation heatmap as jpeg and eps
jpeg("Figure.correlation_plot.jpeg", width = 1200, height = 1200)
corrplot(M,method ='circle',tl.col = "black")
corrplot(
  M,
  method='color',
  type = 'upper',
  add = T ,
  tl.pos = "n",
  cl.pos = "n",
  diag = F,
  p.mat = testRes$p,
  sig.level = c(0.001,0.01,0.05),
  pch.cex = 1.5,
  insig = 'label_sig'
)
dev.off()
```
````
````

#4.Using EBICglasso to estimate the overall network

```
```{r}
groups <- c(
            rep('Cyberchondria (CSS-12)', 12),
            rep('Pandemic fear (FCV-19S)', 7),
            rep('Alexithymia (TAS-20)', 3)
)

node_names <- c(
            "Frequently search symptoms online",
            "Read various web pages on symptoms",
            "Repeatedly search symptoms online",
            "Fine until reading serious conditions online",
            "Increased anxiety after symptom research",
            "Panic reading rare online conditions",
            "Online research leads to consultation",
            "Suggest online-found diagnostic procedures",
            "Online research prompts specialist consultation",
            "Symptom research distracts from online activities",
            "Symptom research interrupts work",
            "Symptom research interrupts social activities",

            "Afraid of COVID-19",
            "Discomfort when thinking about COVID-19",
            "Clammy hands when thinking about COVID-19",
            "Fear of losing life due to COVID-19",
            "Nervousness when watching news about COVID-19",
            "Sleep difficulties due to worry about COVID-19",
            "Palpitations when thinking about COVID-19",

            "Difficulty identifying feelings",
            "Difficulty describing feelings",
            "Externally oriented thinking"
```
```

```
)

network_selected<-estimateNetwork(data_selected, default = "EBICglasso")

# save & plot network
network_selected_g<-qgraph(network_selected$graph, posCol="#7494BC", negCol="#F3A8A8",
                            fade = TRUE, layout="spring", vsize=6, borders=TRUE,
                            legend=TRUE, groups=groups,
                            title= "The overall network",
                            minimum=0.01,
                            color=c( "#BCA7CB","#A0CBEB","#B7DCAA"),
                            nodeNames=node_names,
                            legend.cex=0.33, label.cex=1,
                            width = 8, height = 5, units = "in", res = 300,
                            filetype="jpg", filename = "Figure_The_overall_network")
jpeg(filename = "Figure_The_overall_network.jpg", width = 8, height = 5, units = "in", res = 300)
plot(network_selected_g)
dev.off()

# Extract the partial correlation matrix for the overall network
graph_matrix <- network_selected[["graph"]]
graph_df <- as.data.frame(graph_matrix)
graph_df <- cbind(row_names = rownames(graph_matrix), graph_df)
graph_df
```

##4.1 Using EBICglasso to estimate inter-construct network

```{r}
AA1<-network_selected$graph[1:12,1:12]
BB1<-network_selected$graph[13:19,13:19]
CC1<-network_selected$graph[20:22,20:22]
```

```
AB1<-matrix(0,12,7)
AC1<-matrix(0,12,3)
Matrix11<-cbind(AA1,AB1,AC1)
BA1<-matrix(0,7,12)
BC1<-matrix(0,7,3)
Matrix12<-cbind(BA1,BB1,BC1)
CA1<-matrix(0,3,12)
CB1<-matrix(0,3,7)
Matrix13<-cbind(CA1,CB1,CC1)
matrix_F<-rbind(Matrix11,Matrix12,Matrix13)
matrix_bridge<-network_selected$graph-matrix_F

# save & plot Inter-construct connections network
matrix_bridge_g<-qgraph(matrix_bridge, posCol="#7494BC", negCol="#F3A8A8",
                                fade = TRUE, layout = "spring", vsize = 6, borders =
TRUE,
                                legend = TRUE, groups = groups,
                                title = "The overall network",
                                minimum = 0.01,
                                color = c("#BCA7CB", "#A0CBEB", "#B7DCAA"), node
Names = node_names,
                                legend.cex = 0.28, label.cex = 1,
                                width = 6, height = 5, units = "in", res = 300,
                                filetype="jpg", filename = "Figure_Inter-construct_conn
ections_network")
jpeg(filename = "Figure_Inter-construct_connections_network.jpg", width = 8, height
= 5, units = "in", res = 300)
plot(matrix_bridge_g)
dev.off()
```

```{r}
c_list <- c("Strength","Closeness","Betweenness")
```

````
```

#5.Save & plot centrality parameters
```{r}
jpeg(filename = "Figure_Centrality_indices_for_overall_network.jpg", width = 8, height = 5, units = "in", res = 300)
centralityPlot(network_selected,orderBy="Strength",scale = c("z-scores"),include = c_list)
dev.off()

# Compute and print centrality indexes
centrality_table <- centralityTable(network_selected_g)
betweenness_values <- centrality_table[centrality_table$measure == "Betweenness", c("node","measure","value")]
print(betweenness_values)

Closeness_values <- centrality_table[centrality_table$measure == "Closeness", c("node","measure","value")]
print(Closeness_values)

Strength_values <- centrality_table[centrality_table$measure == "Strength", c("node","measure","value")]
print(Strength_values)
```

#6.Plot bridge strength parameters
```{r}
bridge_symp <- bridge(network_selected$graph, communities=c(
                rep('1', 12),
                rep('2', 7),
                rep('3', 3)
))
````

```
# Plot Bridge_strength figure
jpeg(filename = "Figure_Bridge_strength_centrality_index_for_overall_network.jpg", wid
th = 3, height = 5, units = "in", res = 300)
plot(bridge_symp, order="value",zscore=TRUE,include=c("Bridge Strength"))
dev.off()

# Print Bridge Strength Z-score values
original_bridge_strength <- bridge_symp$`Bridge Strength`
mean_bridge <- mean(original_bridge_strength)
sd_bridge <- sd(original_bridge_strength)
z_scores <- (original_bridge_strength - mean_bridge) / sd_bridge
print(z_scores)
```

#7.Plot boot parameters

```{r}
bootPro_1<-bootnet(network_selected,default =c("EBICglasso"),statistics =
                         c("edge", "strength", "closeness", "betweenness","bridgeStrengt
h"), nBoots=500, nCores=8, communities=groups)
```

```{r}
# Plot Bootstrap for edge
jpeg(filename = "Figure_BootPro_for_edge.jpg",
     width = 12, height = 8, units = "in", res = 300)
par(mar = c(5, 10, 4, 2) + 0.1,
    cex.axis = 0.3,
    las = 2)
plot(bootPro_1, "edge", order = "sample", labels = TRUE,
     cex.label = 0.3,
     label.cex = 0.3)
dev.off()
```

```
# Plot edge weight difference test
jpeg(filename = "Figure_Bootstrapped_Edge_Weights_Difference_Test.jpg",
     width = 10, height = 10, units = "in", res = 300)
plot(bootPro_1, "edge", plot = "difference",
     onlyNonZero = TRUE,
     order = "sample")
dev.off()

jpeg(filename = "Figure_BootPro_for_strength.jpg", width = 8, height = 5, units = "in
", res = 300)
plot(bootPro_1,"strength",plot = "difference", order = "sample")

jpeg(filename = "Figure_BootPro_for_bridgeStrength.jpg", width = 8, height = 5, unit
s = "in", res = 300)
plot(bootPro_1,"bridgeStrength",plot = "difference",order = "sample")
```

#8.Plot CS parameters

```{r}
bootPro_2<-bootnet(network_selected,default =c("EBICglasso"),statistics =
                          c("edge", "strength", "closeness", "betweenness","bridgeStrengt
h"),nBoots=500, type="case", nCores=8,communities=groups)
corStability(bootPro_2)

jpeg(filename = "Figure_CorStabilit_for_strength_bridgeStrength.jpg", width = 8, heigh
t = 5, units = "in", res = 300)
plot(bootPro_2,c("strength", "bridgeStrength"))
dev.off()
```

#9.Compare network stratified by demograpghics

##9.1 Compare edu (1.Junior college and below vs 2/3.Undergraduate and above)

```{r}
```

```
data_edu_yes<-data_selected[which(data$Edu==1),]
data_edu_no<-data_selected[which(data$Edu==2 | data$Edu==3),]

network_edu_yes<-estimateNetwork(data_edu_yes,default = "EBICglasso")
network_edu_no<-estimateNetwork(data_edu_no,default = "EBICglasso")

LL <- averageLayout(network_edu_yes, network_edu_no)

#Plot network_edu_junior_college_and_below
network_edu_yes_g<-qgraph(
                        network_edu_yes$graph,
                        posCol="#7494BC", negCol="#F3A8A8",
                        fade = TRUE, layout=LL, vsize=6, borders=TRUE,
                        legend=TRUE, groups=groups,
                        title= "edu type: junior_college_or_below",
                        minimum=0.01,
                        color=c("#BCA7CB","#A0CBEB","#B7DCAA"),
                        nodeNames=node_names,
                        legend.cex=0.33, label.cex=1,
                        width = 8, height = 5,
                        units = "in", res = 300,
                        filetype="jpg", filename = "Figure_edutype_junior_college_or_belo
w")

#Plot network_edu_undergraduate and above
network_edu_no_g<-qgraph(
                        network_edu_no$graph,
                        posCol="#7494BC", negCol="#F3A8A8",
                        fade = TRUE, layout=LL, vsize=6, borders=TRUE,
                        legend=TRUE, groups=groups,
                        title= "edu type: undergraduate_or_above",
                        minimum=0.01,
                        color=c("#BCA7CB","#A0CBEB","#B7DCAA"),
```

```
                        nodeNames=node_names,
                        legend.cex=0.33, label.cex=1,
                        width = 8, height = 5,
                        units = "in", res = 300,
                        filetype="jpg", filename = "Figure_edutype_undergraduate_or_abo
ve")

# merge to one figure
par(mfrow = c(1, 2))
network_edu_yes_g <- qgraph(network_edu_yes$graph,
                        posCol="#7494BC", negCol="#F3A8A8",
                        fade = TRUE, layout=LL, vsize=6, borders=TRUE,
                        legend=FALSE, groups=groups,
                        title= "Educational level: junior college or below",
                        minimum=0.01,
                        color=c("#BCA7CB","#A0CBEB","#B7DCAA"),
                        nodeNames=node_names,
                        legend.cex=0.33, label.cex=1,
                        width = 8, height = 5, units = "in", res = 300)
network_edu_no_g <- qgraph(network_edu_no$graph,
                        posCol="#7494BC", negCol="#F3A8A8",
                        fade = TRUE, layout=LL, vsize=6, borders=TRUE,
                        legend=FALSE, groups=groups,
                        title= "Educational level: undergraduate or above",
                        minimum=0.01,
                        color=c("#BCA7CB","#A0CBEB","#B7DCAA"),
                        nodeNames=node_names,
                        legend.cex=0.33, label.cex=1,
                        width = 8, height = 5, units = "in", res = 300)
jpeg(filename = "Figure_Combined_Network_Graphs_edutype.jpg", width = 10, height
= 5, units = "in", res = 300)
par(mfrow = c(1, 2))
plot(network_edu_yes_g)
```

````
plot(network_edu_no_g)
dev.off()
par(mfrow = c(1, 1))

#Plot network_edu_yes and network_edu_no Centrality
jpeg(filename = "Figure_Centrality for edutype.jpg", width = 8, height = 5, units = "in", res = 300)
centralityPlot(list(data_edu_yes=network_edu_yes, data_edu_no=network_edu_no), orderBy="Strength",scale = c("z–scores"),include = c_list)
dev.off()
```

```{r}
compare_edu_12<– NCT(data_edu_yes, data_edu_no, binary.data=FALSE, test.edges=TRUE, communities=list,useCommunities="all",
                        test.centrality=TRUE,p.adjust.methods="BH",edges="all")
```

```{r}
#(1)Global Strength Difference
compare_edu_12$glstrinv.sep
compare_edu_12$glstrinv.real
compare_edu_12$glstrinv.pval

#(2)Network Structure Difference
compare_edu_12$nwinv.pval

#(3)Individual Edge Strength Differences
compare_edu_12$einv.pvals

#(4)Difference in node centrality measures
compare_edu_12$diffcen.pval
```
````
````

##9.2 Compare W_yrC (1.1–5 vs 2.6–10 vs 3.11–15 vs 4.≥ 16)

```
```{r}
data_W_yrC_1<-data_selected[which(data$W_yrC==1),]
data_W_yrC_2<-data_selected[which(data$W_yrC==2),]
data_W_yrC_3<-data_selected[which(data$W_yrC==3),]
data_W_yrC_4<-data_selected[which(data$W_yrC==4),]

network_W_yrC_1<-estimateNetwork(data_W_yrC_1,default = "EBICglasso")
network_W_yrC_2<-estimateNetwork(data_W_yrC_2,default = "EBICglasso")
network_W_yrC_3<-estimateNetwork(data_W_yrC_3,default = "EBICglasso")
network_W_yrC_4<-estimateNetwork(data_W_yrC_4,default = "EBICglasso")

LL <- averageLayout(network_W_yrC_1, network_W_yrC_2, network_W_yrC_3, networ
k_W_yrC_4)

#Plot network_W_yrC_1_g
network_W_yrC_1_g<-qgraph(
                    network_W_yrC_1$graph, posCol="#7494BC", negCol="#F3A8A8",
                    fade = TRUE,layout=LL, vsize=6, borders=TRUE,
                    legend=TRUE, groups=groups,
                    title= "1-5 years",
                    minimum=0.01,
                    color=c("#BCA7CB","#A0CBEB","#B7DCAA"),
                    nodeNames=node_names,
                    legend.cex=0.33, label.cex=1,
                    width = 8, height = 5,
                    units = "in", res = 300,
                    filetype="jpg", filename = "Figure_W_yrC_1")

#Plot network_W_yrC_2_g
network_W_yrC_2_g<-qgraph(
                   network_W_yrC_2$graph, posCol="#7494BC", negCol="#F3A8A8",
```
```

```
                    fade = TRUE,layout=LL, vsize=6, borders=TRUE,
                    legend=TRUE, groups=groups,
                    title= "6-10 years",
                    minimum=0.01,
                    color=c("#BCA7CB","#A0CBEB","#B7DCAA"),
                    nodeNames=node_names,
                    legend.cex=0.33, label.cex=1,
                    width = 8, height = 5,
                    units = "in", res = 300,
                    filetype="jpg", filename = "Figure_W_yrC_2")

#Plot network_W_yrC_3_g
network_W_yrC_3_g<-qgraph(
                    network_W_yrC_3$graph,posCol="#7494BC", negCol="#F3A8A8",
                    fade = TRUE,layout=LL, vsize=6, borders=TRUE,
                    legend=TRUE, groups=groups,
                    title= "11-16 years",
                    minimum=0.01,
                    color=c("#BCA7CB","#A0CBEB","#B7DCAA"),
                    nodeNames=node_names,
                    legend.cex=0.33, label.cex=1,
                    width = 8, height = 5,
                    units = "in", res = 300,
                    filetype="jpg", filename = "Figure_W_yrC_3")

#Plot network_W_yrC_4_g
network_W_yrC_4_g<-qgraph(
                    network_W_yrC_4$graph,posCol="#7494BC", negCol="#F3A8A8",
                    fade = TRUE,layout=LL, vsize=6, borders=TRUE,
                    legend=TRUE, groups=groups,
                    title= "≥ 16 years",
                    minimum=0.01,
                    color=c("#BCA7CB","#A0CBEB","#B7DCAA"),
```

```
                    nodeNames=node_names,
                    legend.cex=0.33, label.cex=1,
                    width = 8, height = 5,
                    units = "in", res = 300,
                    filetype="jpg", filename = "Figure_W_yrC_4")

# Merge to one figure
par(mfrow = c(1, 4))
network_W_yrC_1_g <- qgraph(
                    network_W_yrC_1$graph,posCol="#7494BC", negCol="#F3A8A8",
                    fade = TRUE,layout=LL, vsize=6, borders=TRUE,
                    legend=FALSE, groups=groups,
                    title= "Working experience: 1-5 years",
                    minimum=0.01,
                    color=c("#BCA7CB","#A0CBEB","#B7DCAA"),
                    nodeNames=node_names,
                    legend.cex=0.33, label.cex=1,
                    width = 5, height = 5, units = "in", res = 300)
network_W_yrC_2_g <- qgraph(
                    network_W_yrC_2$graph,posCol="#7494BC", negCol="#F3A8A8",
                    fade = TRUE,layout=LL, vsize=6, borders=TRUE,
                    legend=FALSE, groups=groups,
                    title= "Working experience: 6-10 years",
                    minimum=0.01,
                    color=c("#BCA7CB","#A0CBEB","#B7DCAA"),
                    nodeNames=node_names,
                    legend.cex=0.33, label.cex=1,
                    width = 5, height = 5, units = "in", res = 300)
network_W_yrC_3_g <- qgraph(
                    network_W_yrC_3$graph,posCol="#7494BC", negCol="#F3A8A8",
                    fade = TRUE,layout=LL, vsize=6, borders=TRUE,
                    legend=FALSE, groups=groups,
                    title= "Working experience: 11-15 years",
```

```
                        minimum=0.01,
                        color=c("#BCA7CB","#A0CBEB","#B7DCAA"),
                        nodeNames=node_names,
                        legend.cex=0.33, label.cex=1,
                        width = 5, height = 5, units = "in", res = 300)
network_W_yrC_4_g <- qgraph(
                        network_W_yrC_4$graph,posCol="#7494BC", negCol="#F3A8A8",
                        fade = TRUE,layout=LL, vsize=6, borders=TRUE,
                        legend=FALSE, groups=groups,
                        title= "Working experience: ≥16 years",
                        minimum=0.01,
                        color=c("#BCA7CB","#A0CBEB","#B7DCAA"),
                        nodeNames=node_names,
                        legend.cex=0.33, label.cex=1,
                        width = 5, height = 5, units = "in", res = 300)

jpeg(filename = "Figure_Combined_Network_Graphs_W_yrC_type.jpg", width = 10, hei
ght = 7.5, units = "in", res = 300)
par(mfrow = c(2, 2))
plot(network_W_yrC_1_g)
plot(network_W_yrC_2_g)
plot(network_W_yrC_3_g)
plot(network_W_yrC_4_g)
dev.off()
par(mfrow = c(1, 1))

#Plot Centrality_for_W_yrC
jpeg(filename = "Figure_Centrality_for_W_yrC.jpg", width = 8, height = 5, units = "in
", res = 300)
centralityPlot(list(data_W_yrC_1=network_W_yrC_1, data_W_yrC_2=network_W_yrC_2, d
ata_W_yrC_3=network_W_yrC_3, data_W_yrC_4=network_W_yrC_4), orderBy="Strength
",scale = c("z-scores"),include = c_list)
dev.off()
```

```

###9.2.1 1-5 vs. 6-10 years

```{r}
compare_4WyrC_12<- NCT(data_W_yrC_1, data_W_yrC_2, binary.data=FALSE, test.edges=TRUE, communities=list,useCommunities="all",
                       test.centrality=TRUE,p.adjust.methods="BH",edges="all")
```

```{r}
#(1)Global Strength Difference
compare_4WyrC_12$glstrinv.sep
compare_4WyrC_12$glstrinv.real
compare_4WyrC_12$glstrinv.pval
#(2)Network Structure Difference
compare_4WyrC_12$nwinv.pval
#(3)Individual Edge Strength Differences
compare_4WyrC_12$einv.pvals
#(4)Difference in node centrality measures
compare_4WyrC_12$diffcen.pval
```

###9.2.2 1-5 vs. 11-15 years

```{r}
compare_4WyrC_13<- NCT(data_W_yrC_1, data_W_yrC_3, binary.data=FALSE, test.edges=TRUE, communities=list,useCommunities="all",
                       test.centrality=TRUE,p.adjust.methods="BH",edges="all")
```

```{r}
#(1)Global Strength Difference
compare_4WyrC_13$glstrinv.sep
compare_4WyrC_13$glstrinv.real

compare_4WyrC_13$glstrinv.pval
#(2)Network Structure Difference
compare_4WyrC_13$nwinv.pval
#(3)Individual Edge Strength Differences
compare_4WyrC_13$einv.pvals
#(4)Difference in node centrality measures
compare_4WyrC_13$diffcen.pval
```

###9.2.3 1-5 vs. ≥ 16 years

```{r}
compare_4WyrC_14<- NCT(data_W_yrC_1, data_W_yrC_4, binary.data=FALSE, test.edges=TRUE, communities=list,useCommunities="all",
                        test.centrality=TRUE,p.adjust.methods="BH",edges="all")
```

```{r}
#(1)Global Strength Difference
compare_4WyrC_14$glstrinv.sep
compare_4WyrC_14$glstrinv.real
compare_4WyrC_14$glstrinv.pval
#(2)Network Structure Difference
compare_4WyrC_14$nwinv.pval
#(3)Individual Edge Strength Differences
compare_4WyrC_14$einv.pvals
#(4)Difference in node centrality measures
compare_4WyrC_14$diffcen.pval
```

###9.2.4 6-10 vs. 11-15 years

```{r}
compare_4WyrC_23<- NCT(data_W_yrC_2, data_W_yrC_3, binary.data=FALSE, test.edges=TRUE, communities=list,useCommunities="all",

```
                          test.centrality=TRUE,p.adjust.methods="BH",edges="all")
```

```{r}
#(1)Global Strength Difference
compare_4WyrC_23$glstrinv.sep
compare_4WyrC_23$glstrinv.real
compare_4WyrC_23$glstrinv.pval
#(2)Network Structure Difference
compare_4WyrC_23$nwinv.pval
#(3)Individual Edge Strength Differences
compare_4WyrC_23$einv.pvals
#(4)Difference in node centrality measures
compare_4WyrC_23$diffcen.pval
```

###9.2.5 6-10 vs. ≥ 16 years

```{r}
compare_4WyrC_24<- NCT(data_W_yrC_2, data_W_yrC_4, binary.data=FALSE, test.edges=TRUE, communities=list,useCommunities="all",
                          test.centrality=TRUE,p.adjust.methods="BH",edges="all")
```

```{r}
#(1)Global Strength Difference
compare_4WyrC_24$glstrinv.sep
compare_4WyrC_24$glstrinv.real
compare_4WyrC_24$glstrinv.pval
#(2)Network Structure Difference
compare_4WyrC_24$nwinv.pval
#(3)Individual Edge Strength Differences
compare_4WyrC_24$einv.pvals
#(4)Difference in node centrality measures
```

~~~~
compare_4WyrC_24$diffcen.pval
```

###9.2.6 11–15 vs. ≥ 16 years
```{r}
compare_4WyrC_34<- NCT(data_W_yrC_3, data_W_yrC_4, binary.data=FALSE, test.edges=TRUE, communities=list,useCommunities="all",
                              test.centrality=TRUE,p.adjust.methods="BH",edges="all")
```

```{r}
#(1)Global Strength Difference
compare_4WyrC_34$glstrinv.sep
compare_4WyrC_34$glstrinv.real
compare_4WyrC_34$glstrinv.pval
#(2)Network Structure Difference
compare_4WyrC_34$nwinv.pval
#(3)Individual Edge Strength Differences
compare_4WyrC_34$einv.pvals
#(4)Difference in node centrality measures
compare_4WyrC_34$diffcen.pval
```
~~~~
